# Biological conversion of methane to organic molecules: towards a low-carbon bioeconomy

**DOI:** 10.1093/nsr/nwaf547

**Published:** 2025-12-02

**Authors:** Jinyi Qian, Lingling Wang, Liang Guo, Tiantian Chai, Xiulai Chen

**Affiliations:** School of Biotechnology and Key Laboratory of Industrial Biotechnology of Ministry of Education, Jiangnan University, Wuxi 214122, China; School of Biotechnology and Key Laboratory of Industrial Biotechnology of Ministry of Education, Jiangnan University, Wuxi 214122, China; School of Life Science, Shanxi Normal University, Taiyuan 030031, China; School of Biotechnology and Key Laboratory of Industrial Biotechnology of Ministry of Education, Jiangnan University, Wuxi 214122, China; School of Biotechnology and Key Laboratory of Industrial Biotechnology of Ministry of Education, Jiangnan University, Wuxi 214122, China

**Keywords:** methane bioconversion, methanotrophs, methanotrophic microbial cell factories, methane pathways, metabolic engineering

## Abstract

The increasing imperative to mitigate greenhouse gas emissions and foster the transition to a low-carbon bioeconomy has intensified interest in methane bioconversion as a sustainable approach for transforming methane into valuable bioproduction. Although advancements have been made in optimizing methanotrophic pathways to improve bioproduction, significant challenges persist, including methane solubility, bioavailability, and metabolic flexibility, limiting the efficiency of methane bioconversion. This review provides a comprehensive overview of the initiatives aimed at developing next-generation methanotrophic cell factories by overcoming the physiological limitations of natural methanotrophs. We first analyze the metabolic characteristics of methanotrophs for assimilating methane into cellular building blocks. Then, we discuss methane assimilation pathways and their unique characteristics in matter and energy transmission for facilitating the integration of methane into central carbon metabolism. Further, we propose a systematic framework for designing methane-based biomanufacturing to enable low-carbon bioproduction by integrating synthetic biology, metabolic engineering, and systems biology, thereby developing efficient methane assimilation cell factories for producing high-value bioproducts. Finally, we prospect the potential for valorizing methane derived from anthropogenic emissions and renewable sources, while identifying the key challenges and future research directions necessary for advancing a sustainable, low-carbon bioeconomy.

## Teaser

Methane bioconversion technologies transform methane into organic molecules, contributing to the development of a low-carbon bioeconomy.

## INTRODUCTION

Nowadays, environmental issues are becoming increasingly prominent, with the emission of large quantities of greenhouse gases gradually emerging as a major driver of environmental deterioration. Among these gases, methane (CH_4_) is one of the most important non-carbon dioxide greenhouse gases, possessing a warming potential that is 84 times greater than carbon dioxide (CO_2_) over a 20-year period, with annual emissions reaching 50 million metric tons (Mt) [1,2]. Thus, the conversion and utilization of CH_4_ has become imperative. Industry has announced the ‘Global Methane Pledge’ aimed at achieving at least a 30% reduction in anthropogenic CH_4_ emissions by 2030 (based on 2020), which underscores a heightened commitment to mitigating CH_4_ emissions [3,4]. CH_4_ serves as a renewable energy source with dual benefits for the environment and energy. It can be derived from various sources including agricultural residues, urban organic waste, and wastewater through many processes such as anaerobic digestion, biomass gasification, and landfill gas recovery. This process not only facilitates waste valorization, but also aids in mitigating greenhouse gas emissions and environmental pollution [4]. Furthermore, CH_4_ can be used for power generation, heating, or conversion into industrial feedstocks, thereby improving energy utilization efficiency, optimizing the energy structure, and promoting the development of a circular economy. These applications provide crucial support for the establishment of a sustainable energy system [5].

Conventional chemical methods can convert CH_4_ to high-value chemicals and fuels through various reactions. However, these processes consume a lot of energy and have considerable environmental impacts. Most chemical methods, such as oxidation and steam CH_4_ reforming, are well-established and widely applied on an industrial scale. These methods can achieve high energy efficiency through process integration optimization and leveraging existing infrastructure. However, they often necessitate elevated temperatures and pressures, resulting in substantial energy consumption and notable CO_2_ emissions [6]. Additionally, these methods are often complex to operate and involve high equipment costs. Compared with chemical catalysis, the biological conversion of CH_4_ is a more energy-efficient and environmentally friendly method [5]. The process typically operates at ambient temperature, has low energy consumption, and can reduce CO_2_ emissions and alleviate the greenhouse effect. These methods offer the potential to reduce the carbon footprint by circumventing high-temperature processes and integrating them with renewable feedstocks. However, biological approaches are still in the early stages, facing many challenges such as low conversion efficiencies, limited product yields, and difficulties in scaling up processes, rendering them less economically competitive for the large-scale production of bulk chemicals or fuels. Thus, CH_4_ bioconversion holds considerable potential for industrial applications. CH_4_ can be efficiently converted to high-value products such as single-cell proteins (SCPs) [7], biofuels (methanol [8], fatty acids [9]), biodegradable plastics (PHA/PHB) [10,11], and organic acids [12]. This bioconversion of CH_4_ not only reduces reliance on conventional petrochemical resources, but also contributes to reducing greenhouse gas emissions stemming from agriculture, landfills, and fossil fuel extraction. For example, *Methylomonas* sp. DH-1 isolated from a brewery was able to convert CH_4_ to methanol through fed-batch cultivation, achieving methanol production up to 1.34 g/L [13]. The biological conversion of CH_4_ serves crucial environmental and ecological functions by reducing energy consumption, cutting CO_2_ emissions, mitigating the greenhouse effect, and optimizing the carbon cycle. Leveraging microorganisms such as methanotrophs offers a promising approach to effectively reduce substantial CH_4_ emissions generated from agriculture (e.g. livestock manure), landfills, and oil and natural gas extraction. This strategy not only reduces atmospheric CH_4_ levels and alleviates global warming pressure, but also facilitates the recovery of waste greenhouse gases. Further, it is also conducive to optimizing the carbon cycle, promoting the development of green energy and sustainable biomanufacturing, and bringing dual benefits to both industry and environment.

Recently, significant progress has been made in biological CH_4_ utilization, particularly in the exploration of methanotrophs and microbial transformation. Current research on CH_4_ utilization by methanotrophs mainly focuses on enhancing biomass and product synthesis through natural pathways for CH_4_ assimilation, such as *Methylococcus capsulatus* producing isoprene and mevalonate [14,15], *Methylotuvimicrobium alcaliphilum* producing ectoine [16], *Methylosinus* producing polyhydroxybutyrate [17], and *Methylosinus trichosporium* producing 3-hydroxypropionic acid [18]. However, due to limitations in microbial metabolic capacity, cultivation conditions, and available genetic tools, the efficiency of CH_4_ utilization is still limited, thereby posing significant challenges for large-scale applications. For instance, isoprene biosynthesis with *M. capsulatus* was constrained by methane monooxygenase (MMO) in the ribulose monophosphate cycle for CH_4_ oxidation. Currently, the highest reported isoprene production from CH_4_ is only 228.1 mg/L, which was achieved by engineering *M. capsulatus* Bath through the introduction of a heterogenous mevalonate (MVA) pathway using a CRISPR-based editing tool [14]. Genetic engineering and metabolic engineering strategies can be employed to modify *M. capsulatus* utilizing CRISPR gene editing tools for inhibiting the expression of the toxic soluble MMO, thereby redirecting carbon flux for bioproduction and improving CH_4_ conversion efficiency [14]. In addition, the integration of CH_4_ assimilation pathways into conventional model microorganisms such as *E. coli* warrants consideration, due to their well-established gene-editing tools and comprehensive understanding of metabolic regulation, thereby facilitating the modular construction and systematic optimization of CH_4_ assimilation pathways [19]. Moreover, their rapid growth and efficient experimental platforms offer advantages for the identification of key enzymatic components and the validation of pathway feasibility. However, the introduction of CH_4_ assimilation pathways into heterologous hosts still faces significant challenges, including the limited solubility of CH_4_, potential limitations in the folding and activity of heterologous enzymes in *E. coli*, imbalances in energy and reducing power between the introduced pathway and the metabolic network of the host strain, and the inability to express the key enzymes for methane oxidation such as CH_4_ monooxygenase in heterologous systems. If all these issues are solved, the full potential of CH_4_ bioconversion would be tapped for the efficient low-carbon biomanufacturing of high-value products.

**Figure 1.**
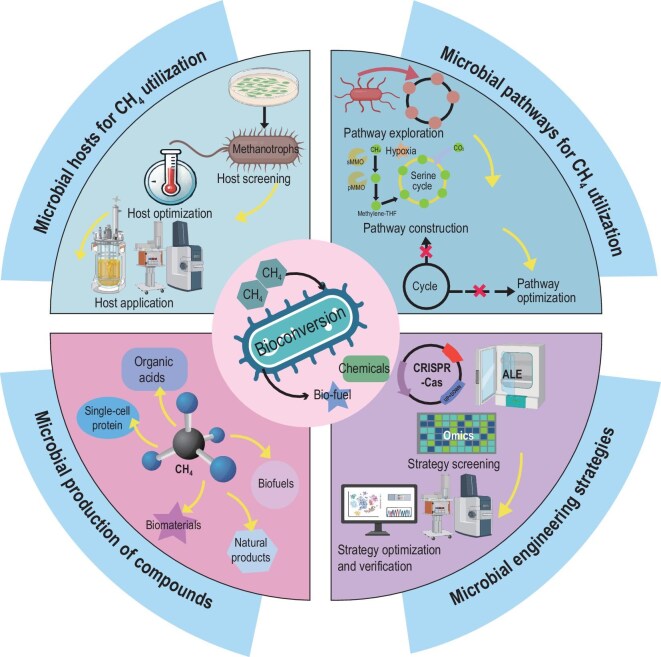
Bioconversion of CH_4_. CH_4_ can be converted into various products, including biochemicals, biofuels, and pharmaceuticals through CH_4_ assimilation pathways in various methanotrophs. The screening and optimization of methanotrophs, the exploration and construction of CH_4_-utilizing pathways, and the development and verification of metabolic engineering and synthetic biology strategies are all keys to enhancing the efficiency of CH_4_ bioconversion.

At present, there is a lack of systematic reviews focusing specifically on CH_4_ bioconversion. The most recent review, published in 2021, mainly describes the conversion of CH_4_ to bioproducts by methanotrophs [20]. Although some reviews on methanol bioconversion briefly mention CH_4_ utilization, they do not provide a comprehensive overview [21]. To comprehensively explore the recent progress of CH_4_ bioconversion, our review focuses on the key challenges encountered by methanotrophs during the bioconversion process, along with potential solutions from three aspects (Fig. 1). First, we systematically summarize three major groups of natural CH_4_-utilizing methanotrophs and analyze their unique metabolic characteristics. Then, we introduce CH_4_ assimilation pathways reported to date and analyze their characteristics and applications for effective and efficient CH_4_ conversion. Next, we discuss microbial engineering strategies to improve the efficiency of CH_4_ utilization and their applications for the low-carbon biomanufacturing of high-value products. Finally, we highlight future trends and key challenges in CH_4_ bioconversion for the future advancement of a low-carbon bioeconomy.

## MICROBIAL HOSTS FOR METHANE UTILIZATION

**Figure 2.**
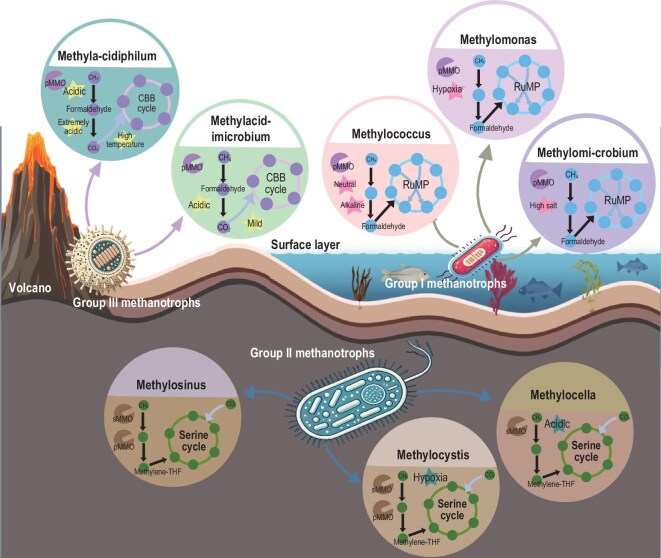
Microbial hosts for CH_4_ utilization. Group I methanotrophs are mainly distributed in aerobic environments, such as wet freshwater, oceans, and wetlands. Group II methanotrophs are mainly distributed in microaerobic environments, such as plant rhizospheres and soils. Group III methanotrophs are specially adapted to extreme environments, such as acidic, volcanic soils, and hot springs.

Methanotrophs are gram-negative bacteria capable of utilizing CH_4_ as both a carbon and energy source. These microorganisms are typically found in environments such as sewage, swamps, and marine sediments and play a vital role in the CH_4_ cycle. Methanotrophs are currently classified into three main groups: Group I, Group II, and Group III, which use the ribulose phosphate pathway, the serine cycle, and the Calvin-Benson-Bassham (CBB) cycle, respectively, to convert CH_4_ into biomass or other metabolites (Table 1 and Fig. 2). Therefore, methanotrophs have broad application potential in environmental protection and industrial biotechnology, and play an important role in mitigating the greenhouse effect.

**Table 1.** Classification and characteristics of methanotrophs.

	Gammaproteobacteria	Alphaproteobacteria	Verrucomicrobia
Pathways	The RuMP cycle	The serine cycle	The CBB cycle
Enzyme system	pMMO/sMMO	pMMO	pMMO
Growth conditions	• Neutral pH • Mesophilic • Moderate O_2_/CH_4_	• Broad pH range • Some nitrogen fixation	• Acidophilic, low pH (2–3) • Thermophilic, high temperature (45–60°C)
Natural habitat	• Freshwater or marine sediments • Wastewater treatment and biogas facilities • Coverings such as oil fields, coal mines, and landfills	• Soil • Plant rhizosphere • Anoxic wetland environments	• Extreme environments, such as hot springs, acidic soils, and volcanic regions
Genetic tractability	Limited but improving	Moderate, some tools available	Limited
Product profile	Natural products, SCPs, organic acids	PHAs, lipids, biopolymers	Part of lipids and polysaccharides
Industrial applicability	Pharmaceuticals, nutritional fortification, food	Biodegradable plastics, cosmetics, medical and biomedical materials, agricultural and environmental applications	Functional foods, cosmetics, and personal care
Pros and cons	• High carbon fixation efficiency • Low ATP consumption • Fast growth rate • Needing high oxygen concentration • Limiting cell growth in hypoxic environments	• Adapting to low CH_4_ concentration and low oxygen environment • High ATP consumption • Slow growth rate	• Tolerating extreme environments, such as hot springs and acid pits • Slow growth rate

### Group I methanotrophs

Group I methanotrophs primarily include γ-proteobacteria, which belong to *Methylococcaceae*. This group encompasses CH_4_-oxidizing bacteria (MOB) [22]. These bacteria assimilate CH_4_ through the ribulose monophosphate pathway and are predominantly found in environments such as freshwater and seawater sediments, wastewater treatment facilities, biogas plants, and areas like oil fields, coal mines, and landfill sites. Currently, Gammaproteobacteria mainly include *Methylococcus, Methylomonas, Methylomicrobium, Methylobacter, Methylocaldum*, etc. [20,23]. These methanotrophs can convert CH_4_ into a variety of high-value-added chemicals, presenting significant practical and economic potential. Consequently, in recent years, research on CH_4_ utilization using methanotrophs as host strains has garnered widespread interest.

#### Methylococcus

*Methylococcus* is a gram-negative aerobic microorganism that uses oxygen as the terminal electron acceptor. It utilizes CH_4_ as its sole source of carbon and energy, playing a critical role in the biosphere, particularly in the CH_4_ cycle. The most common and widely studied methanotroph in *Methylococcus* is *Methylococcus capsulatus*, whose metabolic pathway strictly depends on MMO for CH_4_ oxidation. It can efficiently utilize high concentrations of CH_4_ in the environment and convert it into organic matter or other high-value-added products, such as SCPs and biofuels [24,25]. When the exogenous mevalonate (MVA) pathway was introduced into *M. capsulatus* Bath, isoprene production reached 228.1 mg/L [14]. However, compared with conventional engineered bacteria, the growth conditions and genetic tools of *M. capsulatus* are limited, especially the extremely limited genetic engineering toolkit. To address this issue, a highly efficient genome editing system for *M. capsulatus* was developed and employed to express exogenous genes such as *mvaE* (acetyl-CoA acetyltransferase/HMG-CoA reductase), *mvaS* (HMG-CoA synthase), *pta* (phosphate acetyltransferase), *fxpk* (fructose-6-phosphate/xylulose-5-phosphate phosphoketolase), and *phaA* (β-ketothiolase) under a phenol-inducible promoter, achieving a maximum mevalonate concentration of 2090 mg/L [15]. Future advancements in transformation tools and genetic engineering methods can broaden the industrial application potential of *M. capsulatus* and provide a viable strategy for CH_4_ bioconversion and environmental sustainability.

#### Methylomonas

*Methylomonas*, a genus within the family *Methylococcaceae* of the class Gammaproteobacteria [26], comprises strictly aerobic bacteria that utilize CH_4_ or methanol as their sole carbon and energy source [27]. Similar to *Methylococcus, Methylomonas* also holds significant biotechnological potential and can produce SCPs. For example, its biomass containing carotenoids is richer than that of *Methylococcus* [28]. *Methylomonas* sp. ZR1 has been isolated for its ability to synthesize C_40_ lycopene, C_30_ carotenoids, and exopolysaccharides from CH_4_ [28,29]. Additionally, under O_2_ levels below 4%, *Methylomonas* sp. DH-1 can produce H_2_ with CH_4_ as the sole carbon source, and this process remains unaffected by CH_4_ content in shake flasks [30]. By deleting succinate dehydrogenase (*sdh*) in the tricarboxylic acid (TCA) cycle, succinate production was increased by 10-fold compared to that of the wild type [31]. Furthermore, an automated genetic engineering system based on Cre-lox was employed to engineer *Methylomonas* sp. DH-1 for the efficient conversion of CH_4_ to squalene, and thus squalene production reached 31.3 mg/L [32]. In addition, laboratory adaptive evolution has also been employed to improve the tolerance of *Methylomonas* sp. DH-1 to lactic acid, followed by metabolic engineering and fermentation optimization strategies that increased lactic acid production to 0.245 g/g CH_4_ [33]. However, its strictly aerobic nature and reliance on CH_4_ or methanol as the sole carbon and energy source may limit biomass accumulation under oxygen-deficient conditions. In addition, limitations in metabolic flux through the TCA cycle and the availability of cofactors may constrain the production of target metabolites such as carotenoids, squalene, and lactic acid. Strategies such as oxygen-controlled cultivation, pathway optimization, and cofactor balancing are expected to further enhance product yields and expand the biotechnological applications of *Methylomonas*.

#### Methylomicrobium

*Methylomicrobium* is an obligate aerobic methanotroph that uses CH_4_ or methanol as its carbon and energy source and is widely distributed in freshwater and marine habitats. It plays a crucial role in CH_4_ oxidation and carbon cycling, with significant potential for biotechnological applications. Based on metabolic characteristics, *Methylomicrobium* can be classified into two groups: obligate methanotrophs and facultative methanotrophs. The obligate methanotrophs can only utilize CH_4_ or methanol as their carbon and energy source, such as *Methylomicrobium alcaliphilum* [34] and *Methylomicrobium album* [35]. By knocking out the *ectD* (ectoine hydroxylase) and *ectR* (MarR-like transcription regulator) genes in *M. alcaliphilum* 20Z, ectoine production reached 142.32 mg/L using CH_4_ as the sole carbon source [16]. Moreover, metabolic engineering of *M. alcaliphilum* 20Z allowed it to consume both CH_4_ and lignocellulose-derived sugars such as glucose and xylose, to produce ectoine, by which ectoine production was increased by 1.7 times compared to that of CH_4_ alone [36]. Facultative methanotrophs can use other small organic molecules (such as formate and acetate) as carbon sources when CH_4_ concentrations are low, such as *Methylomicrobium buryatense* [37]. *M. buryatense* 5GB1 has been shown to grow primarily on CH_4_, while also utilizing other substrates like acetate [38]. This metabolic flexibility offers promising opportunities for the industrial application of CH_4_ in combination with other carbon sources to produce high-value-added products, and provides new ideas and directions for improving the efficiency of CH_4_ bioconversion.

#### Others

In addition to the three species mentioned above, *Methylobacter* [39,40], *Methylosarcina* [9,41], and *Methylohalobius* [42,43] are also classified as Group I methanotrophic bacteria, but these genera have been relatively less studied. For *Methylosarcina* sp. LC-4, the Plackett–Burman statistical method was used to evaluate the positive effects of trace elements such as nitrate, phosphate, and tungstate in the culture medium on the synthesis of fatty acid methyl esters using CH_4_. Through adjusting the composition of the culture medium, the production of fatty acid methyl esters was increased by 85.7% compared to the unoptimized state [9]. On the other hand, most strains are still in the stage of species identification and analysis of the environment and mechanism, such as *Methylohalobius* [44]. This type of strain is specially adapted to extreme environments such as saline-alkali land, salt lakes, and salt marshes, and plays an important role in CH_4_ degradation in these ecosystems. *Methylohalobius crimeensis* was discovered in high-salt environments [42,43], and its unique physiological characteristics, along with its extreme habitat, make it a valuable model for potential industrial applications.

### Group II methanotrophs

Group II methanotrophs primarily include α-proteobacteria, which belong to *Methylocystaceae*. This group represents one of the most significant and extensively studied bacterial categories [45], including photoautotrophic bacteria, plant symbiotic bacteria (such as *Rhizobium*), endosymbiotic bacteria (such as *Wolbachia*), and intracellular parasitic bacteria (such as *Rickettsiella*). Among them, some of these microorganisms can use CH_4_ as a carbon and energy source to synthesize various metabolites. α-Proteobacteria primarily include *Methylosinus, Methylocystis, Methylocella*, and *Methylocapsa*, which can assimilate CH_4_ through the serine cycle [23].

#### Methylosinus

*Methylosinus* is a significant genus of aerobic, gram-negative methanotrophic bacteria belonging to the α-proteobacteria group that utilizes CH_4_ as a carbon and energy source. These bacteria play a vital role in the CH_4_ cycle by oxidizing CH_4_ to CO_2_, which is predominantly found in soils, wetlands, and freshwater ecosystems. Currently, the most extensively studied species in this genus is *Methylosinus trichosporium*, which oxidizes CH_4_ to methanol via soluble CH_4_ monooxygenase (sMMO) and membrane-bound CH_4_ monooxygenase (pMMO), and then converts it to CO_2_ or uses it for biosynthesis through a series of metabolic pathways [46]. For instance, the malonyl-CoA pathway in *M. trichosporium* OB3b was reconstructed by heterologously expressing a bifunctional enzyme, malonyl-CoA reductase from *Chloroflexus aurantiacus*, resulting in a 3-hydroxypropionic acid production of 60.59 mg/L [18]. Additionally, this species has also been engineered to produce 251.5 mg/L of (R)-1,2-propanediol from epoxide [47]. Further, a genome-scale metabolic network model, *i*MsOB3b, for *M. trichosporium* OB3b has been developed and used to analyze the distribution of carbon flux between central metabolic pathways and CH_4_ bioconversion [48]. This model provides a foundation for systematically exploring the CH_4_ metabolic mechanism and pointing out the direction for future research on CH_4_ utilization.

#### Methylocystis

*Methylocystis* is a type of methanotroph that utilizes strict aerobic respiratory metabolism, depending on oxygen as the terminal electron acceptor and using CH_4_ and methanol as their carbon and energy sources [49]. This genus has been detected in various environments, including wetlands [50], glacial foreland soil [51], lakes [52], and other places where CH_4_ exists. They have significant potential in producing valuable bioproducts from CH_4_, including polyhydroxybutyrate (PHB) [24,53,54], poly(3-hydroxybutyrate-co-3-hydroxyvalerate) [55], and phytoene [56]. For example, elevated CO₂ concentrations during the cultivation of *Methylocystis* on CH_4_ have been demonstrated to enhance PHB production by 1.4- to 1.6-fold compared to culture on CH_4_ alone [57]. Similarly, the ratio of O_2_ to CH_4_ significantly affects PHB production. The elevated O_2_ (O_2_/CH_4_ ratio of 1:5) enhanced biomass productivity by 1.51-fold during the same interval compared with that of low O_2_ conditions [58]. At present, *Methylocystis* is underexploited in the realm of metabolic engineering, primarily due to the limited availability of genetic manipulation tools and the complexity of metabolic networks. Through a stepwise metabolic engineering approach, the biosynthesis pathway of phytoene was constructed and optimized in *Methylocystis* sp. MJC1, with the production of phytoene in fed-batch fermentation reaching 15 mg/L [56]. Additionally, the CRISPR/Cas9 genome editing system in *Methylocystis parvus* OBBP has provided a convenient tool for gene deletion and insertion, laying a solid foundation for advancing CH_4_ bioconversion [50]. However, the CRISPR/Cas9 gene editing system, while significant, is insufficient on its own. Thus, the development of additional genetic manipulation tools suitable for CH_4_ utilization is presently crucial for enhancing the efficiency of CH_4_ conversion.

#### Methylocella

*Methylocella* is a gram-negative, aerobic methanotroph capable of utilizing CH_4_ as well as multi-carbon compounds, such as monomethylamine and acetate. As a facultative methanotroph, it can be isolated from acidic environments, including swamps, forest soils, and tundra wetlands [51,52]. They are abundant and metabolically active in continental gas seeps, playing an important role in the biogeochemical cycling of natural gas alkanes [59]. To date, three *Methylocella* species capable of utilizing CH_4_ have been discovered, namely *Methylocella silvestris* [60], *Methylocella palustris* [61], and *Methylocella tundrae* [62]. However, research on these bacteria remains limited, mostly focusing on fermentation optimization. For example, the optimization of culture conditions for *M. tundrae* during the bioconversion of CH_4_ to methanol resulted in an increase in methanol production from 0.66 mM to 5.18 mM [63]. Due to the lack of advanced genetic engineering tools, it is difficult to optimize the production of biochemicals through metabolic engineering in *Methylocella*. Developing genetic modification techniques will be pivotal to fully unlocking the industrial potential of *Methylocella*. Future research should focus on developing precise genetic engineering tools for *Methylocella*, such as CRISPR-Cas systems, to enable precise enhancement of key metabolic pathways. In parallel, comprehensive multi-omics analyses including transcriptomics, proteomics, and metabolomics should be applied to elucidate its CH_4_ and multi-carbon assimilation networks, thereby providing a basis for identifying core regulatory nodes and metabolic bottlenecks. Such integrated strategies are expected to fully unlock the industrial potential of *Methylocella*, positioning it as an efficient microbial platform for the sustainable production of fuels and high-value chemicals.

#### Others

*Alphaproteobacteria* are comprised of fewer species compared to Gammaproteobacteria, leading to a relatively diminished focus in research endeavors. In addition to the three aforementioned methanotrophs, *Methylocapsa* emerges as another group II methanotroph capable of utilizing CH_4_. Predominantly inhabiting acidic soils and wetlands, *Methylocapsa* significantly contributes to the process of CH_4_ oxidation. To date, only three bacterial species have been identified: *Methylocapsa acidiphila* [64], *Methylocapsa gorgona* [65], and *Methylocapsa palsarum* [66]. Research has primarily focused on their growth environments and metabolic mechanisms, leaving genetic or metabolic engineering largely unexplored. Notably, the acid-tolerant methanotroph *M. acidiphila* possesses the remarkable ability to produce SCPs from biogas, thereby offering new technological insights into waste-to-protein conversion [67]. This technology opens new prospects for *Methylocapsa* research and lays a foundation for optimizing CH_4_ utilization in future studies.

### Group III methanotrophs

Group III methanotrophs primarily consist of Verrucomicrobia, which are commonly found in acidic geothermal ecosystems and assimilate CH_4_ through the Calvin-Benson-Bassham (CBB) cycle [68–71]. These bacteria are capable of thriving in acidic environments, with some species tolerating a minimum pH as low as 1.0 [72]. To date, only two bacterial genera associated with CH_4_ oxidation have been investigated, including *Methylacidiphilum* and *Methylacidimicrobium* [23].

#### Methylacidiphilum

*Methylacidiphilum* is a genus of bacteria within the phylum Verrucomicrobia, including species such as *Methylacidiphilum infernorum* [73], *Methylacidiphilum kamchatkense* [74], and *Methylacidiphilum fumariolicum* [75]. These bacteria are capable of thriving in extreme environments, such as volcanic and geothermal areas [76], where they can oxidize sulfide and CH_4_ simultaneously [77]. Methyl mercaptan inhibits CH_4_ oxidation, but *M. fumariolicum* SolV has a unique ability to consume methyl mercaptan to generate H_2_S, which it then oxidizes. This process facilitates the oxidation and utilization of CH_4_ [78]. Moreover, *M. fumariolicum* SolV can also facilitate the conversion of CH_4_ to methanol. By optimizing its growth medium, the methanol production rate reached 0.88 mM/gDCW/h [79]. Currently, research on these bacteria mainly focuses on fundamental metabolism, with limited research on genetic modification and metabolic engineering. In addition, due to their extreme growth conditions, additional investigation is necessary to broaden the application of *Methylacidiphilum* in industrial biotransformation.

#### Methylacidimicrobium

*Methylacidimicrobium* is a distinct group of methanotrophic bacteria belonging to the phylum Verrucomicrobia. There are two main species: *Methylacidimicrobium tartarophylax* and *Methylacidimicrobium thermophilum*, both of which thrive in extremely acidic and volcanic environments [80]. These microorganisms utilize CH_4_ as their primary energy and carbon source, playing a crucial role in understanding the biogeochemical cycle of CH_4_. Currently, research on *Methylacidimicrobium* is limited, with most studies focusing on genomic analysis. Despite their scientific importance, these bacteria have not yet been widely explored for applications in metabolic engineering, resulting in untapped biotechnological potential. Further exploration of their metabolic pathways may yield novel insights and practical applications in CH_4_ bioconversion.

### Anaerobic methanotrophs

Anaerobic methanotrophs (ANME) are capable of oxidizing CH_4_ under anaerobic conditions, often forming symbiotic relationships with non–CH_4_-oxidizing bacteria. They are primarily found in water-saturated environments. In contrast to aerobic methanotrophs that rely on CH_4_ monooxygenase, ANME oxidize methane to CO_2_ through the reverse methanogenesis pathway, using electron acceptors such as sulfur and nitrogen compounds or high-valence metal ions like iron and manganese [81]. Phylogenetically, ANME are categorized into lineages, including ANME-1, ANME-2, and ANME-3 [81–83]. They exhibit slow growth rates, with doubling times extending over several tens of days, and are adapted to low-energy, sulfide-rich, or high-pressure environments. Additionally, they possess unique archaeal lipids, such as archaeol and hydroxyarchaeol, which confer thermal stability [83,84]. ANME play a critical role in the global carbon cycle by mitigating CH_4_ emissions in anaerobic environments such as sediments. By converting CH_4_, a potent greenhouse gas, into biomass, organic acids, or high-value lipids, and coupling this process to energy generation under specific conditions, ANME hold significant potential for applications in CH_4_ valorization, environmental remediation, microbial fuel cells, and as microbial cell factories for the production of biofuels, biosurfactants, and thermally resilient biomaterials.

Anaerobic oxidation of CH_4_ (AOM) is a key process in the global CH_4_ cycle and can be classified into several types based on the utilized electron acceptors. The earliest discovered type is sulfate-driven anaerobic CH_4_ oxidation (SAMO), facilitated by the symbiotic relationship between anaerobic methanotrophic archaea (ANME-1) and sulfate-reducing bacteria (SRB) [85]. This process mainly occurs in anaerobic environments such as marine sediments and is estimated to account for ∼90% of CH_4_ consumption in the oceans [86,87]. Its metabolic mechanism has been confirmed to follow the reverse methanogenesis pathway, in which methane is oxidized to CO_2_ and SO_4_^2−^ is reduced to HS^−^ [88]. Denitrifying anaerobic CH_4_ oxidation (DAMO) mainly includes nitrate-driven (ANME-2d) and nitrite-driven (NC_10_) processes [83]. The nitrate-driven process, similar to SAMO, follows the reverse methanogenesis pathway, but use a different electron acceptor: NO^3−^ is reduced to NO^2−^. In contrast, during the nitrite-driven AOM, nitrite is ultimately reduced to N_2_ and O_2_, where the generated oxygen facilitates CH_4_ oxidation [89]. Apart from sulfate and nitrogen oxides, metal-mediated CH_4_ oxidation plays a significant role in sulfur-deficient deep-sea environments. Fe^3+^ and Mn^4+^ can serve as terminal electron acceptors for oxidizing CH_4_ to CO_2_ [90]. The mechanisms of extracellular electron transfer (EET) are currently under investigation and are believed to involve three potential pathways: (i) direct electron transfer from the inner membrane to the extracellular electron acceptor via outer membrane cytochromes; (ii) transfer via conductive pili extending from the outer membrane; and (iii) secretion of redox-active mediators that facilitate electron shuttling between the cell and electron acceptor. Additionally, metal ions such as Se^6+^, Cr^6+^, As^5+^, V^5+^, Sb^5+^, and Te^4+^ have been demonstrated to mediate anaerobic CH_4_ oxidation [91]. Overall, these processes differ in electron acceptors and electron transfer mechanisms, but all rely on the reverse methanogenesis pathway to activate and oxidize CH_4_. They not only reveal the close interactions of CH_4_ with sulfur, nitrogen, and metal cycles, but also provide valuable insights for greenhouse gas mitigation and methane resource utilization.

## MICROBIAL PATHWAYS FOR METHANE UTILIZATION

**Figure 3.**
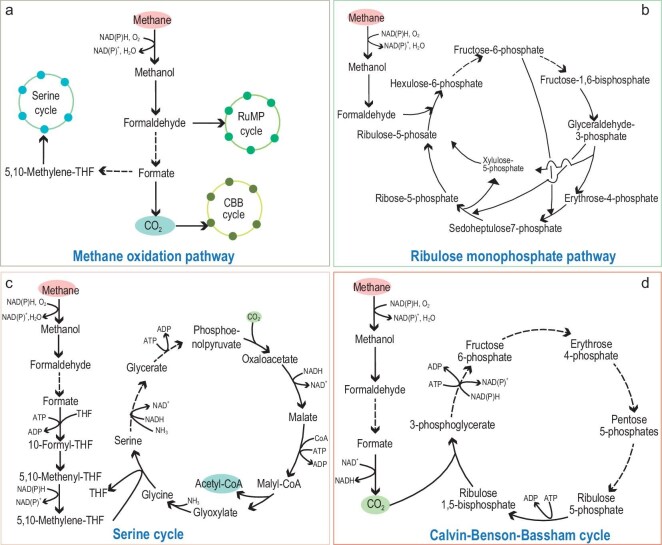
Microbial pathways for CH_4_ utilization. (a) Methane oxidation pathway; (b) the RuMP cycle; (c) the serine cycle; (d) the CBB cycle. The red balls represent CH_4_, the green balls represent CO_2_, and the blue balls represent intermediate metabolites.

Methanotrophs are capable of utilizing CH_4_ as both an energy and carbon source, converting CH_4_ to various metabolites through specific metabolic pathways. Methanotrophs utilize CH_4_ primarily through two interconnected pathways: CH_4_ oxidation and CH_4_ assimilation. CH_4_ oxidation constitutes the initial and essential step in C_1_ compound metabolism. CH_4_ assimilation predominantly occurs via three distinct metabolic routes: the ribulose monophosphate (RuMP) pathway, the serine cycle, and the Calvin-Benson-Bassham (CBB) cycle (Table 2 and Fig. 3) [92]. These pathways play a crucial role in mitigating atmospheric CH_4_ levels.

**Table 2.** Comparison of methane assimilation pathways.

	The RuMP cycle	The serine cycle	The CBB cycle
ATP consumption	1	3	7
NAD(P)H consumption	1	4	4
Carbon conversion efficiency	High (60%–70%)	Medium (30%–50%)	Low (25%–40%)
Thermodynamic efficiency	High thermodynamic efficiency with low ATP demand	Moderate thermodynamic efficiency, but high reducing equivalent demand	Low thermodynamic efficiency due to high ATP and NADPH requirements
Industrial potential	• Suitable for efficient methanol-to-chemicals conversion (amino acids, organic acids, etc.) • Compatible with synthetic methylotrophic *E. coli* • Scalable for industrial applications	• Promising for complex metabolite production from C_1_ feedstocks • Holding potential for extended application in systems metabolic engineering	• Supporting CO_2_-based carbon-neutral biomanufacturing • Allowing introduction into heterotrophic hosts via metabolic engineering • Contributing to sustainable bioproduction
Key enzymes and their stability/expression challenges	• 3-hexulose-6-phosphate synthase (Moderate; presenting difficulties in achieving high soluble expression in heterologous hosts) • 6-phospho-3-hexuloisomerase (Generally stable; low expression in heterologous hosts)	• Serine hydroxymethyltransferase (Moderate; causing toxicity at high expression levels; requiring proper cofactor) • Malyl-CoA lyase (Moderate; sensitive to temperature/pH; low soluble expression in heterologous hosts)	• Rubisco (Low intrinsic stability; presenting difficulties in folding and assembling in heterologous hosts) • Phosphoribulokinase (Generally stable; requiring co-expression with Rubisco)

### Methane oxidation for C1 metabolism

The CH_4_ oxidation pathway is a sequential process through which methanotrophic bacteria convert CH_4_ to CO_2_ and H_2_O under aerobic conditions (Fig. 3a). It is a preliminary step common to all methanotrophs. This pathway is extensively present in nature, thriving in habitats such as soils, wetlands, and the aerobic layers of lakes. It not only plays an indispensable role in the global carbon cycle, but also possesses considerable environmental significance due to its capacity to regulate the potent greenhouse gas CH_4_. The oxidative pathway of methanotrophic bacteria primarily relies on the presence of their unique methane monooxygenase (MMO), which uses O_2_ as the terminal electron acceptor to oxidize CH_4_ into methanol [93]. As the key initial enzyme in this process, MMO can be classified into two types: soluble MMO (sMMO) and particulate MMO (pMMO). sMMO is typically located in the cytoplasm and is encoded by the *mmoXYBZDC* operon, with its expression occurring under conditions of low copper concentrations (<0.005 mol/L) [94]. In contrast, pMMO is typically located in the intracytoplasmic membrane, where it is either membrane-bound or partially associated with the membrane. The encoding of pMMO is attributed to the *pmoCAB* operon, which comprises genes *pmoB, pmoA*, and *pmoC*, corresponding to the polypeptides that form the α, β, and γ subunits of pMMO, respectively [95]. Copper ions function as transcriptional activators of pMMO, with the expression levels of pMMO exhibiting an increase in response to elevated concentrations of copper ions [96]. Most methanotrophic bacteria are characterized by the presence of pMMO, while *Methylocella* [61] and *Methyloferula* [97] exclusively express sMMO. Additionally, certain strains, including *Methylococcus capsulatus* [96] and *Methylosinus trichosporium* [98], have the capability to express both sMMO and pMMO. Following the initial step, methanol undergoes further oxidation to formaldehyde through the action of a pyrroloquinoline quinone (PQQ)-dependent methanol dehydrogenase. The initial oxidation of CH_4_ requires energy input in the form of NADH, while the energy required for the subsequent oxidation processes is derived from these initial reactions. In this oxidative sequence, formaldehyde is converted to formate by formaldehyde dehydrogenase, which is subsequently oxidized to CO_2_ by formate dehydrogenase. This oxidative pathway is a crucial component of the biological carbon cycle and also contributes to the mitigation of CH_4_, a greenhouse gas with a high global warming potential.

### The RuMP cycle

The ribulose monophosphate (RuMP) cycle is widely distributed not only among methylotrophic bacteria but also across various other bacterial and archaeal species (Fig. 3b) [99]. It serves as a key metabolic pathway that enables microorganisms to assimilate one-carbon compounds and plays an essential role in the conversion of CH_4_ to metabolites in methanotrophs. In these bacteria, CH_4_ is converted to methanol catalyzed by MMO, then oxidized to formaldehyde, and ultimately assimilated into the central carbon metabolism via the RuMP cycle [21,100]. In the RuMP cycle, there are three main stages: carbon fixation, carbon cleavage, and carbon rearrangement. In the carbon fixation stage, formaldehyde condenses with ribulose 5-phosphate, catalyzed by 3-hexulose-6-phosphate synthase, forming hexulose 6-phosphate. Hexulose 6-phosphate is then converted into fructose 6-phosphate by the action of hexose phosphate isomerase. In the carbon cleavage stage, fructose 6-phosphate is split into glyceraldehyde 3-phosphate and dihydroxyacetone phosphate, catalyzed by a series of enzymes in the glycolysis pathway. In the carbon rearrangement stage, fructose 6-phosphate and glyceraldehyde 3-phosphate are condensed to form xylose-5-phosphate and erythrose 4-phosphate via transketolase. Then, erythrose 4-phosphate and fructose 6-phosphate are condensed to form sedoheptulose-7-phosphate by transaldolase. Sedoheptulose-7-phosphate is next combined with glyceraldehyde 3-phosphate to form xylose-5-phosphate and ribose-5-phosphate, which are converted to ribulose 5-phosphate. Compared to other pathways, the RuMP cycle is recognized as the most energy-efficient option [21], so this pathway is commonly used for improving the growth rate of microbial cells and increasing the efficiency of CH_4_ conversion.

### The serine cycle

The serine cycle demonstrates how methanotrophic bacteria utilize CH_4_ as a carbon and energy source for biotransformation through a sophisticated metabolic network (Fig. 3c). This pathway is crucial for understanding the effective utilization of CH_4_ [92]. MMO catalyzes the conversion of CH_4_ to methanol, which is oxidized to formaldehyde and then to formate by formaldehyde dehydrogenase. Formate is first converted to methyltetrahydrofolate by a series of enzymes, and then methyltetrahydrofolate and glycine are catalyzed to synthesize serine by serine hydroxymethyltransferase (SHMT). Serine is next converted to hydroxypyruvate, which is converted to 2 molecules of 2-phosphoglycerate by NADH-dependent hydroxypyruvate reductase and glycerate kinase. One molecule of 2-phosphoglycerate is assimilated into the downstream pathway, while the other molecule of 2-phosphoglycerate is further converted into phosphoenolpyruvate, catalyzed by enolase. Phosphoenolpyruvate is carboxylated to generate oxaloacetate, followed by malic acid and malyl-CoA. Malyl-CoA is then converted into glyoxylic acid and acetyl-CoA by the action of malyl-CoA lyase. Finally, glycine is condensed with glyoxylic acid to complete the cycle. However, the formation of 1 molecule of acetyl-CoA by the serine cycle theoretically requires 2 molecules of NAD(P)H and 3 molecules of ATP, rendering it less favorable as a pathway for achieving efficient biological utilization of CH_4_ [101]. Although the serine cycles can be applied to methanotrophs, this process has not been widely implemented in other microorganisms due to the special structural properties of the enzymes involved in the conversion of CH_4_ to formate [102]. Thus, future development of the serine cycle for CH_4_ utilization will focus on a comprehensive investigation of the expression and regulation of key enzymes to enhance the efficiency of CH_4_ bioconversion. For example, MMO plays a crucial role in influencing the efficiency of CH_4_ bioconversion by catalyzing the initial oxidation of CH_4_ to methanol. However, its low catalytic rate and substrate specificity pose significant challenges. To address these issues, genetic engineering strategies can be used to enhance enzyme expression, modify the catalytic active center of key enzymes for improving catalytic efficiency, or introduce more efficient enzyme variants sourced from other organisms. Additionally, metabolic engineering can be applied to redirect carbon fluxes towards target products, while systems biology methodologies, such as genome-scale metabolic modeling and omics analyses, can offer systematic approaches for pinpointing metabolic bottlenecks. Furthermore, laboratory adaptive evolution alongside high-throughput screening technologies will facilitate the identification of more efficient strains. By integrating these strategies, enzymatic steps and overall metabolic networks can be optimized, thereby improving the efficiency of CH_4_-to-product conversion and advancing the development of sustainable biomanufacturing.

### The CBB cycle

The Calvin-Benson-Bassham (CBB) cycle represents a prominent pathway for CO_2_ assimilation, commonly found in plants, algae, cyanobacteria, and certain autotrophic bacteria and archaea (Fig. 3d). This cycle serves as the main carbon consumption pathway on earth, driving >99.5% of the ∼120 billion tons of carbon to sugars by plants, algae, and cyanobacteria [103]. The CBB cycle recycles ATP and NADPH generated by the light reaction in photosynthesis, serving as energy and reducing force power. It captures CO_2_ in the atmosphere and converts it to C_3_ compounds in cells through a series of enzymatic reactions. This cycle is fundamental for life on earth to synthesize organic matter, and can be engineered and optimized to efficiently produce the C_2_ compound acetyl-CoA [104]. In Group III methanotrophs, formate is converted to CO_2_ by NAD^+^-dependent formate dehydrogenase, and then the assimilation of CH_4_ as a single carbon source is realized through the CBB cycle, utilizing CO_2_ as a key substrate [105]. Ribulose-1,5-bisphosphate carboxylase oxygenase (RuBisCO), a key enzyme in the CBB cycle, is also functionally expressed in various microorganisms. For example, in *Methylacidiphilum fumariolicum* SolV, the enzyme facilitates the incorporation of CO_2_ into the CBB cycle, thereby enabling the assimilation of CH_4_ as a single carbon source through the integration of CO_2_ and 1,5-bisphosphate ribulose. The bacteria could grow with CO_2_ as the sole carbon source and use CH_4_ as energy to synthesize metabolites [106]. Here, RuBisCO plays a pivotal role in affecting the efficiency of CH_4_ bioconversion. In methanotrophs, RuBisCO is responsible for carbon fixation, but its limited turnover rate constrains the overall efficiency of carbon assimilation. Additionally, the metabolic network of the CBB cycle can be deeply analyzed using systems biology and metabolic engineering methods to find new regulatory targets and pathways to improve the efficiency of CH_4_ bioconversion, which is of great significance for promoting the sustainable utilization of CH_4_ resources and is an important part of advancing CH_4_-based clean energy and carbon capture technology.

## MICROBIAL ENGINEERING STRATEGIES FOR METHANE UTILIZATION

**Figure 4.**
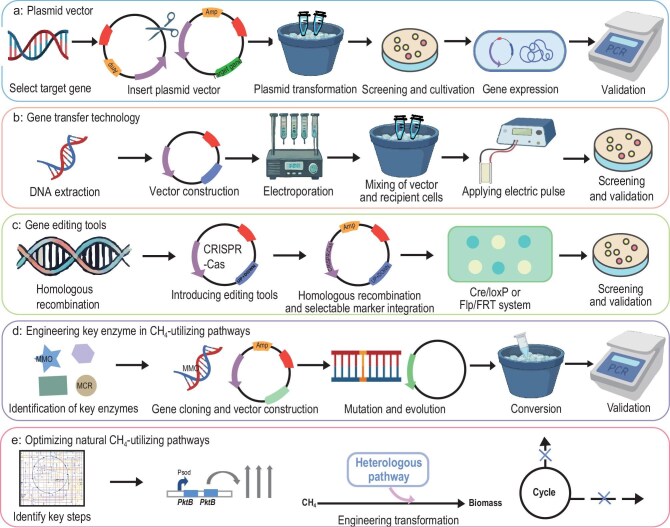
Genetic and metabolic engineering strategies for constructing CH_4_-utilizing cell factories. (a) Plasmid vectors; (b) gene transfer technology; (c) gene editing tools; (d) engineering key enzymes in CH_4_-utilizing pathways; (e) optimizing natural CH_4_-utilizing pathways. Amp: ampicillin.

**Figure 5.**
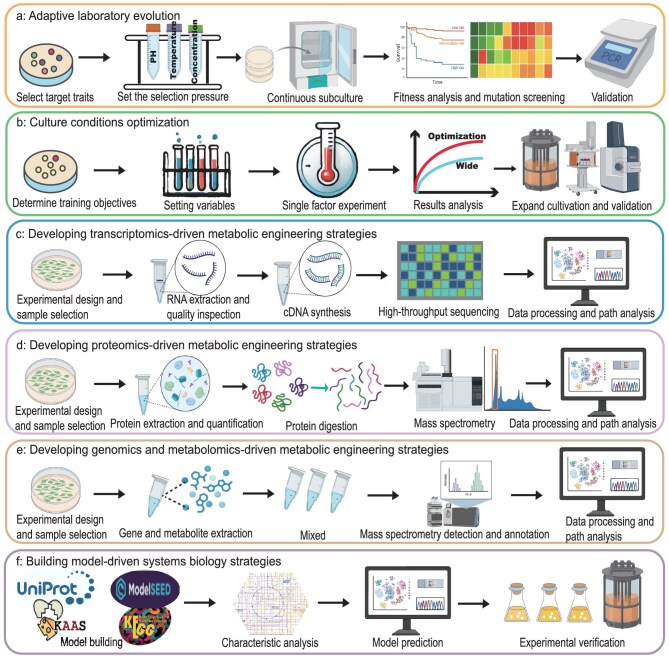
Systems and omics-driven strategies for developing CH_4_-utilizing cell factories. (a) Adaptive laboratory evolution; (b) culture conditions optimization; (c) transcriptomics-driven metabolic engineering strategies; (d) proteomics-driven metabolic engineering strategies; (e) developing genomics- and metabolomics-driven metabolic engineering strategies; (f) building model-driven systems biology strategies.

Microbial engineering strategies are methodologies used to modify and optimize microorganisms for specific industrial, environmental, or medical applications. These strategies leverage the inherent metabolic capabilities of microorganisms and enhance them through genetic and metabolic engineering techniques. Microbial engineering strategies for CH_4_ utilization focus on elevating the efficiency of microbial CH_4_ assimilation and thus increasing product yield. These strategies primarily encompass tools for genetic modification, pathways for CH_4_ utilization, the engineering of CH_4_-utilizing hosts, omics-driven strategies for metabolic engineering, and model-driven systems biology strategies (Figs 4 and 5).

### Constructing genetic tools

The development of genetic modification tools is an important field in microbial engineering for CH_4_ utilization, especially in the research and application of methanotrophs. Methanotrophs possess the remarkable capability for converting CH_4_ to methanol through their unique MMOs, which represents the initial step in CH_4_ metabolism. To improve the efficiency of CH_4_ bioconversion, researchers have developed a variety of genetic modification tools and strategies, including gene expression elements, plasmid vectors, gene transfer technology, and gene editing tools.

#### Gene expression elements

The expression level of a gene is typically influenced by regulatory elements such as promoters, ribosome binding sites (RBS), and terminators, which play a central role in controlling the expression of key metabolic enzymes. Since promoters and RBS are located in the upstream functional regions of the target gene, they serve as crucial *cis*-regulatory elements for transcriptional and translational regulation. Therefore, by replacing promoters or RBS of varying strengths, the expression levels of MMO and its downstream metabolic enzymes can be effectively enhanced, thereby accelerating the conversion of CH_4_ to methanol and subsequent metabolites. For example, by screening various promoters, including inducible and constitutive types, ribosome binding sites RBS1, RBS2, RBS3, and RBS4, and plasmid vectors, three distinct expression constructs pTETR, pCMR, and pAMR were developed in *Methylomicrobium buryatense* 5GB1. Through optimization of these component combinations, the engineered *M. buryatense* 5GB1 strain achieved a lactic acid titer of 0.6 g/L [107]. The introduction of the inducer anhydrotetracycline modifies the conformation of the tetracycline repressor protein, thereby alleviating the binding inhibition between the inducible promoter P_tetR_ and RNA polymerase. This mechanism allows for regulating gene expression for lactic acid biosynthesis in *M. buryatense*, resulting in a maximum production of 1.3 g/L [108]. Furthermore, the phenol-inducible promoter P_O_ has been widely applied in the metabolic engineering of *M. capsulatus* Bath for the high-level production of compounds such as hydroxymethylglutarate and isoprene [14,15]. Promoters commonly found in methanotrophs include constitutive promoters (P_mxaF_, P_tac_, P_trc_) and inducible promoters (P_tetR_, PPO), exhibiting varying strengths by 10–100-fold [109]. RBS selection similarly affects translation initiation efficiency with sequences of varying strengths generally identified through the combination of *in silico* prediction and experimental screening. Despite these efforts, the array of *cis*-regulatory elements in methanotrophs remains limited, and a systematic, quantitative database of promoter strengths is still lacking. Future work should integrate transcriptomic analyses with synthetic biology to establish a reliable regulatory framework for precise control of metabolic flux. Overall, the precise regulation of expression elements not only enhances the CH_4_ metabolic capacity of methanotrophs but also provides strong support for the development of innovative CH_4_ conversion platforms within the realm of synthetic biology. This advancement holds significant implications for advancing the industrial application of CH_4_ bioconversion.

#### Plasmid vectors

Plasmid vectors serve as indispensable tools for the genetic modification of CH_4_ bioconversion, playing a crucial role in the replication, expression, and regulation of genes related to CH_4_ metabolism via small circular DNA molecules (Fig. 4a). They are pivotal in achieving efficient gene expression and hold substantial significance in improving the efficiency of CH_4_ conversion. In CH_4_ bioconversion, plasmid vectors have two important functions: (1) enhancing the efficient expression of target genes can improve the ability of microorganisms to utilize CH_4_. Using *M. buryatense* 5GB1 as a host capable of converting CH_4_ to lactic acid, three different combinations of expression vectors (pTETR, pCMR, and pAMR) were constructed by screening various promoters, ribosome binding sites, and plasmid vectors. Following a process of combined optimization, lactic acid production was increased by 14-fold compared with previous reports in a bioreactor system [107,108]. (2) Facilitating the incorporation of heterologous genes to reconstruct the CH_4_ metabolic pathway, thereby improving conversion efficiency. For example, by introducing heterologous genes *budA* (α-acetolactate decarboxylase from *Klebsiella pneumoniae*) and *budB* (acetolactate synthase from *Bacillus subtilis*) into *Methylomicrobium alcaliphilum* 20Z using plasmid vectors, the synthesis pathway of 2,3-butanediol was constructed and optimized, achieving a titer of 68.8 mg/L [110]. Frequently used plasmid systems include vectors derived from origins of replication such as RSF1010, pBBR1, and pAWP89, generally with medium copy numbers (∼10–20) [111]. However, some vectors exhibit inadequate stability in different hosts, and the limited availability of selectable markers pose challenges for multi-gene or multi-module expression. Future efforts should focus on developing customizable, modular plasmid systems, optimizing plasmid performance through high-throughput screening, and exploring chromosome-integrating vectors to enhance genetic stability. Additionally, plasmid vectors can also enhance gene function, impart new metabolic capabilities to microorganisms, and drive advancements in efficient CH_4_ bioconversion.

#### Gene transfer technology

Gene transfer technology is a genetic modification tool that introduces exogenous genes into microbial cells to alter their genetic information (Fig. 4b). This technology plays a key role in the research and application of methanotrophic bacteria, especially in improving the efficiency of CH_4_ conversion and producing high-value-added products. This is primarily accomplished through conjugation and electroporation technology [112]. Conjugation transfer involves coupling methanotrophic bacteria with other bacteria to facilitate the transfer of plasmid vectors. For example, plasmid vectors incorporating RK2/RP4 transfer origins can be transferred from *E. coli* S17-1 into methanotrophic bacteria through conjugation, achieving genetic material transformation by the process of mating [23]. Electroporation technology enables plasmids to penetrate the cell membranes of methanotrophic bacteria by creating temporary pores through the application of high-voltage electric fields. For example, the plasmid vector pAWP89, extracted from *E. coli*, can be introduced into *Methylocystis iwaonis* SD4 under CH_4_ conditions via electroporation (1500 V, 25 μF, and 150 Ω) with a frequency of ∼9 × 10^6^ CFU/μg DNA. Subsequently, *M. iwaonis* SD4 is allowed to recover in NMS liquid medium under CH_4_ conditions, achieving a high transformation efficiency [113]. The effective application of this rapid and efficient gene transfer technology in methanotrophic bacteria has facilitated successful gene transfer, providing a valuable strategy for rapid CH_4_ conversion and significantly advancing the transformation and optimization of microorganisms related to CH_4_ metabolism. Plasmid systems commonly utilized in methanotrophs derived from broad–host-range origins such as RSF1010, RK2/RP4, and pBBR1, are predominantly characterized by copy numbers ranging from 5 to 20 and demonstrate consistent maintenance across diverse genera [111]. However, replication efficiency and compatibility with different hosts can vary significantly, thereby limiting the applicability of genetic modifications. Future work should aim to develop modular, broad-host-range vectors and optimize electroporation and conjugation protocols to enhance the efficiency and stability of plasmid transfer.

#### Gene editing tools

Gene editing tools are a class of molecular technologies that enable precise insertion, deletion, or replacement of DNA sequences at specific loci within the genome (Fig. 4c). Common examples include scarless gene knockout and integration techniques, the CRISPR/Cas9 system, and the Cre-lox recombination system. These tools allow for the regulation of key enzymes and metabolic pathway-related genes in methanotrophs, thereby optimizing microbial metabolic networks and significantly enhancing CH_4_ conversion efficiency and the yield of target products. Scarless gene knockout and integration technology is a crucial tool for achieving precise genome editing. It enables the deletion of target genes or the insertion of exogenous genes without leaving exogenous markers or selective markers. Targeted genome modification in methanotrophic bacteria is of great significance for understanding their biological characteristics and advancing biotechnology applications. For example, the sucrose lethal gene *sacB* (levansucrase) counterselection system based on scarless gene knockout was validated by knocking out glycogen production and assessing promoter strength in *M. buryatense* [111]. In addition, in *Methylotuvimicrobium buryatense* 5GB1C, scarless gene knockout based on the *pheS* (phenylalanyl-tRNA synthetase beta subunit) counterselectable marker greatly improved the gene knockout efficiency, with a positive rate of >92% and a shortened operation time of 8 days [114]. Furthermore, the Cre-lox system can be utilized in *M. buryatense* 5GB1C to construct a CRISPRi library for the screening of essential genes [115]. Thus, in the process of CH_4_ bioconversion, the use of scarless gene knockout and integration technology, along with the Cre-lox recombination system, allows for regulating the gene expression of methanotrophic bacteria, optimizing the metabolic pathway of CH_4_ assimilation, improving the efficiency of CH_4_ utilization, and minimizing unnecessary genetic traces. The application of this technology provides robust support for increasing product yield and advancing industrial applications in CH_4_ bioconversion. The CRISPR/Cas9 editing system is an RNA-guided gene editing tool derived from the bacterial immune defense mechanism. It uses a single-guide RNA (sgRNA) to direct the Cas9 nuclease to specific loci in the genome, where it introduces double-strand breaks, enabling targeted gene knockout, insertion, or site-specific mutation. The CRISPR/Cas9 editing system was first established in *M. capsulatus* Bath, achieving a gene knockout efficiency of up to 71% [116]. Furthermore, the CRISPR/Cas9 system facilitates the knockout of sMMO and the modification of green fluorescent protein in *M. capsulatus* Bath [116]. The CRISPR/Cas9 system also has certain limitations, such as off-target effects, resulting in unintended gene editing or undesired genetic alterations. To improve the efficiency of gene editing, future directions should involve implementing novel nucleases with high specificity, advancing CRISPRi/a systems for reversible regulation of gene expression, and incorporating Cre-lox systems to establish a versatile editing framework. Overall, the advancement of gene editing technologies has not only enhanced our comprehension of CH_4_ metabolism but also offered substantial support for the establishment of effective microbial platforms for CH_4_ conversion. This progress contributes to the sustainable utilization of CH_4_ resources and fosters improvements in environmental management.

### Optimizing CH_4_-utilizing pathways

In the study of methanotrophic bacteria, the optimization of the CH_4_ utilization pathway emerges as a pivotal strategy for enhancing CH_4_ conversion efficiency. By precisely controlling carbon flux in this pathway, it is possible to boost conversion efficiency and facilitate the efficient synthesis of target products, thereby rationally constructing microbial cell factories. However, to achieve effective control over carbon flux, the optimization of CH_4_ fixation enzymes and assimilation pathways represents a promising approach.

#### Engineering the key enzyme in CH_4_-utilizing pathways

MMO and methyl-coenzyme M reductase (MCR) are the only two enzymes known for their natural ability to oxidize CH_4_ (Fig. 4d). MMO, utilizing oxygen as an electron acceptor oxidant, primarily converts CH_4_ to methanol, laying the foundation for subsequent metabolic activities. However, MMO faces some limitations, such as low activity, inadequate stability, and high requirements for oxygen, which hinder its bioconversion efficiency. To address these challenges, various strategies have been developed to enhance the application potential of MMO. For example, with *M. alcaliphilum* 20Z lacking methanol dehydrogenase as the host strain, formate addition could increase the activity of MMO, with methanol titer and productivity reaching 2.15 g/L and 0.717 g/L/h, respectively [117]. MCR mainly catalyzes the conversion of CH_4_ to CO_2_ under anaerobic conditions, typically in CH_4_-rich but oxygen-poor environments such as marine sediments, lake bottoms, and wetlands. MCR is highly dependent on coenzymes and has low catalytic efficiency, posing challenges for industrial production. For example, MCR is dependent on coenzyme F430, and the purification of the enzyme required to synthesize coenzyme F430 subsequently enhances MCR activity [118]. Current research primarily focuses on understanding the catalytic structure and function of MCR, with few efforts in enhancing its catalytic efficiency. Therefore, in the future, gene editing, high-throughput screening technology, and laboratory adaptive evolution could be employed to improve the catalytic efficiency of MCR and boost the efficiency of CH_4_ bioconversion.

#### Optimizing natural CH_4_-utilizing pathways

Optimizing natural CH_4_-utilizing pathways stands as a crucial strategy to improve the efficiency of CH_4_ bioconversion, reduce energy consumption and greenhouse gas emissions, and promote a green and low-carbon economy (Fig. 4e). However, the complexity and uncertainty of microbial metabolic pathways pose significant challenges to the optimization process of natural CH_4_-utilizing pathways. Thus, to address these limitations, two main strategies have been proposed. On the one hand, genetic engineering can be used to modify key enzymes in the CH_4_-utilizing pathway, systematically reconstructing the metabolic network, and minimizing the accumulation of by-products. For example, in *M. buryatense*, overexpression of phosphoketolase (PktB) in the phosphoketolase pathway led to a 2-fold increase in acetyl-CoA concentration and a 2.6-fold and 2-fold improvement in CH_4_ to microbial biomass and lipid yields compared to those of the wild-type strain, respectively [119]. On the other hand, systems metabolic engineering can be used to integrate CH_4_ metabolism with heterologous biosynthetic pathways to efficiently synthesize target products. For example, metabolic engineering was conducted on the non-oxidative segment of the pentose phosphate pathway in the ribulose monophosphate (RuMP) cycle in *M. alcaliphilum* 20Z, and this was integrated with the xylose utilization pathway to achieve the production of shinorine up to 17.13 mg/L [120]. These results demonstrate that by engineering specific carbon flux ‘valves’ (such as phosphoketolase, transketolase, and transaldolase), the assimilation efficiency of CH_4_-derived carbon can be significantly improved. Despite the promising strategies for regulating carbon flow and modifying metabolic pathways, there still exist many challenges, such as insufficient genetic tools and complex metabolic networks, hindering further industrial advancement. By intensifying the analysis of CH_4_ metabolic networks and enhancing the development of genetic tools, the development of a CH_4_ bioeconomy can be significantly propelled.

Faced with the limitations of insufficient genetic tools and low conversion rates in natural CH_4_-utilizing pathways, the modification of traditional microbial cell factories emerges as a promising solution. The introduction of heterologous genes and pathways is poised to become a crucial strategy for constructing non-natural pathways, which are committed to improving CH_4_ bioconversion efficiency and advancing its industrial application. However, the distinctive structure of MMO and its tightly controlled CH_4_ oxidation mechanism pose challenges for its expression in various hosts. This is primarily due to its complex multi-subunit structure and specific cofactor requirements, thereby hindering the development of non-natural methanotrophic cell factories [121]. Future progress relies on the development of adaptable CRISPR editing tools, broadening the range of CH_4_-activating enzyme libraries, and implementing rationally designed adaptive laboratory evolution (ALE) to further optimize metabolic flux balance. These strategies will accelerate the construction of efficient CH_4_-utilizing cell factories and their integration into industrial applications.

### Engineering CH₄-utilizing hosts

Optimizing microbial cell factories serves as an effective approach for regulating cell adaptability to CH_4_, thereby enabling microorganisms to tolerate and utilize this substrate more efficiently. This process typically involves various strategies, such as ALE and the optimization of culture conditions, aimed at selecting strains with increased CH_4_ tolerance. Consequently, this leads to the more efficient conversion of CH_4_ to valuable products, such as biofuels or chemicals.

#### Adaptive laboratory evolution

ALE enables the selection of superior mutants by imposing specific environmental pressures, thereby enhancing microbial adaptability, metabolic efficiency, and production capacity (Fig. 5a). Selective pressures, such as high concentrations of target products, pH fluctuations, or nutrient limitations, can enrich mutants with enhanced tolerance or metabolic efficiency under stress conditions. In CH_4_-utilizing microorganisms, ALE can improve CH_4_ assimilation, increase product tolerance, and optimize metabolic fluxes regulated by key enzymes. For example, ALE can be utilized to screen tolerant strains. ALE was used to screen the lactic acid-tolerant strain *Methylomonas* sp. DH-1 JHM80. When combined with gene expression regulation strategies, lactic acid titer reached 1.19 g/L with its yield up to 0.245 g/g CH_4_ [33]. Additionally, ALE can enhance cell tolerance to products, thereby improving CH_4_ conversion efficiency. Given that rhamnolipids exert an inhibitory effect on the growth of *M. alcaliphilum*, ALE enabled the strain to thrive in an environment containing 5 g/L rhamnolipids, thereby enhancing the conversion of CH_4_ to rhamnolipids [122]. Thus, ALE holds great promise for accelerating microbial evolution and advancing the CH_4_ bioeconomy.

#### Culture conditions optimization

In addition to ALE, optimizing culture conditions represents another key strategy to improve the adaptability of methanogenic cells (Fig. 5b). Appropriate culture conditions are crucial for increasing cell biomass, improving CH_4_ utilization efficiency, and elevating metabolite production. In this context, optimization efforts can be divided into two parts: refining culture substrates and facilitating co-cultivation. The first is to optimize the culture substrate, which encompasses not only the formulation of the culture medium but also the surrounding environment. For instance, *M. fumariolicum* SolV is used to produce methanol, but it is incapable of synthesizing methanol at pH 3.0. Only at pH 5.5 does the strain begin methanol production [79]. Thus, adjusting pH can effectively enhance CH_4_ conversion into target metabolites. Second, co-culturing methanotrophs with other bacteria can significantly boost CH_4_ conversion efficiency. By integrating complementary metabolic functions, co-culture systems can surpass the inherent limitations of individual microorganisms. For example, co-culturing *M. capsulatus* Bath and *E. coli* SBA01 for CH_4_ conversion into mevalonate resulted in its production up to 61 mg/L using CH_4_ as the sole carbon source [123]. Such division of labor allows for efficient carbon utilization while minimizing the accumulation of inhibitory intermediate metabolites. Moreover, co-culture systems offer distinct advantages under anaerobic or microaerobic conditions, where CH_4_ conversion is often constrained by gas–liquid mass transfer and energy efficiency. Pairing methanotrophs with fermentative or photosynthetic microorganisms can synergistically enhance electron transfer, maintain redox balance, and stabilize the overall metabolic network. Recent studies have further demonstrated that co-culture systems enhance process robustness against environmental fluctuations, thereby supporting the sustainable operation of industrial bioreactors [124]. Therefore, optimizing culture conditions represents a multifaceted strategy that involves not only the selection of the culture substrate but also other critical factors such as the cultural environment. A comprehensive approach to these optimized conditions can significantly enhance product yield and facilitate the transition towards industrial application.

### Developing omics-driven metabolic engineering strategies

With the advancement of various biotechnologies, omics-driven metabolic engineering strategies have gained increasing attention. This approach entails a comprehensive analysis of the genes, enzymes, and metabolic pathways of microorganisms by integrating multiple omics technologies such as genomics, transcriptomics, proteomics, and metabolomics, etc. Such analysis helps to elucidate the mechanisms of CH_4_ metabolism, optimize the pathways for CH_4_ conversion, and improve CH_4_ utilization efficiency and product yield, thereby illuminating the direction for biological CH_4_ conversion.

#### Transcriptomics

Transcriptomics plays a critical role in CH_4_ utilization by providing a comprehensive analysis of gene expression regulation and metabolic mechanisms (Fig. 5c). By analyzing the transcriptome of methanotrophic bacteria, key enzymes, regulatory factors, and metabolic networks involved in CH_4_ bioconversion can be identified, thereby offering valuable insights for optimizing CH_4_ conversion processes. First, the application of transcriptomics can effectively improve the synthesis of target products. For example, transcriptomics studies on fatty acid biosynthesis in *Methylomicrobium buratense* 5G (B1) revealed that fatty acid degradation, as well as the availability of acetyl-CoA and malonyl-CoA, are bottlenecks for increasing fatty acid production. Through genetic engineering technology, following the knockout of acetate kinase and the overexpression of acetyl-CoA carboxylase, the accumulation of fatty acids reached 111 mg/gDCW, representing a 20% increase compared with that of the original strain [125]. Additionally, transcriptomics analysis can be used to optimize culture conditions to promote the growth of methanotrophic bacteria. For example, the effect of the CH_4_ and O_2_ supply ratio in *M. buratense* 5GB1 was analyzed by transcriptomics. When the CH_4_/O_2_ supply molar ratio was 0.93, the highest growth rate was obtained, reaching 0.287/h [126]. Thus, transcriptomics provides a systems biology perspective to explore the metabolic mechanism of methanotrophic bacteria, offering new research directions and industrial applications in the efficient bioconversion of CH_4_ and the synthesis of valuable metabolites.

#### Proteomics

Proteomics technology enables the analysis of proteins expressed by microorganisms under specific conditions, thereby uncovering the dynamic fluctuations of key enzymes and metabolic pathways (Fig. 5d). This research is valuable for revealing the metabolic activities associated with CH_4_ oxidation and plays a crucial role in optimizing CH_4_ conversion efficiency. For instance, proteomic analysis revealed that *Methylocystis* sp. strain SC2 could increase biomass yield to 13.82 mgDCW/mmol CH_4_ under oxygen-limited conditions by utilizing hydrogenase, which consequently allows for more CH_4_ utilization in fermentation production [127]. Additionally, proteomic data, combined with transcriptomics and metabolomics, can be used to explore the mechanism for enhancing the cell growth and carbon transport of *M. alcaliphilum* 20Z. Proteomic analysis showed that under culture conditions containing lanthanum, there was a reduction in the intermediates of the ribulose-monophosphate (RuMP) pathway, while the metabolites of the TCA cycle showed an increase [128]. In this way, combining various types of omics data can significantly deepen the understanding of methanotrophic bacteria from the perspective of systems biology, and are beneficial to analyzing the regulatory mechanism of gene expression and the metabolic process of methanotrophic bacteria, thereby advancing the bioconversion of CH_4_.

#### Genomics and metabolomics

With the advancement of omics technologies, although the application of genomics and metabolomics in CH_4_ bioconversion remains limited, they play a critical role in the study of methanotrophs (Fig. 5e). Genomics enables the analysis of the complete genome sequence of methanotrophs, allowing for the identification of key genes, enzymes, and regulatory networks involved in CH_4_ metabolism, thus providing a genetic blueprint for understanding how these organisms utilize CH_4_. Meanwhile, metabolomics allows for the systematic analysis of metabolites produced under different conditions, revealing dynamic metabolic changes during CH_4_ conversion and helping to identify active pathways and potential bottlenecks in CH_4_ utilization efficiency. The first genomic-level study on methanotrophs was reported in 2004, in which the complete genome sequence of *M. capsulatus* Bath was analyzed using genomic technologies, revealing a copper-responsive regulatory mechanism [129]. Since then, an increasing number of methanotroph genomes have been published, such as *M. trichosporium* OB3b [130], *M. silvestris* BL2 [131], and *M. alcaliphilum* 20Z [132]. These genomic resources offer a theoretical foundation for a more comprehensive understanding of the metabolic mechanisms associated with CH_4_ conversion. For example, by further integrating metabolomics with ^13^C-labeling experiments, researchers conducted an in-depth analysis of the central C1 metabolic pathways in *M. trichosporium* OB3b, such as the serine cycle, EMC pathway, and TCA cycle, and confirmed the accumulation of intermediate metabolites, including methanol, formaldehyde, and formate [133]. Therefore, the combination of genomics and metabolomics facilitates the precise engineering and metabolic regulation of methanotrophs. This approach establishes a research framework aimed at enhancing the conversion efficiency of CH_4_ into high-value products, thereby advancing the effective and sustainable utilization of CH_4_ resources.

**Figure 6.**
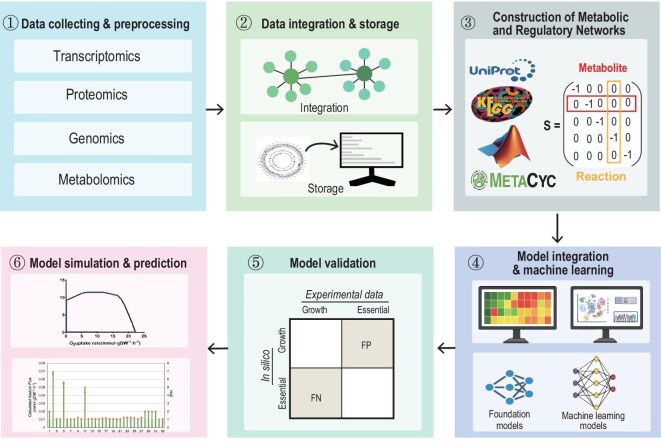
A framework for multi-omics integration. This framework illustrates the workflow from multi-omics data acquisition to predictive modeling. (1) Data collection and preprocessing of transcriptomics, proteomics, genomics, and metabolomics data; (2) data integration and storage; (3) construction of metabolic and regulatory networks using databases such as UniProt, KEGG, and MetaCyc; (4) model integration and machine learning for comprehensive analysis; (5) model validation with experimental data such as cell growth and gene essentiality; and (6) model simulation and prediction for system-level insights.

The increasing application of omics technologies in metabolic engineering and synthetic biology has underscored the importance of translating multi-level omics data into practical metabolic engineering strategies. Integration of multi-omics datasets, including genomics, transcriptomics, proteomics, and metabolomics is crucial for identifying potential targets for metabolic engineering (Fig. 6). Standardization of omics data and the construction of metabolic and regulatory networks facilitate the elucidation of functional associations and regulatory connections among genes, enzymes, and metabolites. Further applying statistical modeling and machine learning methods enables the identification of key nodes and the prediction of their effects on metabolic fluxes. Subsequently, potential targets are prioritized based on prediction performance, robustness, and experimental feasibility, and their effectiveness is ultimately confirmed through experimental validation. This integrated omics strategy facilitates the conversion of descriptive omics data into actionable metabolic engineering schemes, providing systematic guidance for optimizing metabolic pathways such as CH_4_ utilization.

### Designing model-driven systems biology strategies

The genome-scale metabolic model (GEM) serves as a pivotal tool in systems biology, encompassing all genes, enzymes, biochemical reactions, and metabolites associated with metabolism in an organism. This integration facilitates the development of a detailed and intricate metabolic network (Fig. 5f). As a core framework for delineating gene–protein–reaction (GPR) relationships, GEM possesses significant capabilities for the quantitative analysis and systematic comprehension of cellular metabolic processes. When combined with various computational algorithms such as flux balance analysis (FBA), flux variability analysis (FVA), and flux sensitivity analysis (FSA), GEM not only effectively simulates the effects of specific environmental conditions on methanotrophs, but also optimizes cultivation parameters and predicts genetic engineering targets. This functionality ultimately supports targeted modifications of microbial strains.

In the field of CH_4_ bioconversion, GEMs have proven to be instrumental in enhancing the efficiency of CH_4_ utilization. To date, a total of 13 models have been developed specifically for research pertaining to CH_4_ metabolism (Table 3). These models facilitate the systematic identification of key genes and metabolic pathways that affect the efficiency of CH_4_ oxidation or assimilation, thereby guiding strain engineering efforts through genetic modifications aimed at improving carbon conversion performance. For example, the model *i*IA407, designed for *M. alcaliphilum* 20Z, was developed to analyze the intracellular carbon flux distribution associated with CH_4_ and methanol metabolism [34]. Based on the *i*IA407 model, a revised version of the *M. alcaliphilum* 20Z model (*i*20ZR-BDO) was developed. By integrating model-based predictions with metabolic engineering approaches, researchers successfully developed a triple-knockout mutant deficient in *ldh* (lactate dehydrogenase), *ack* (acetate kinase), and *mdh* (malate dehydrogenase). This engineered strain demonstrated a marked increase in product accumulation under oxygen-limited conditions, achieving a final concentration of 2,3-butanediol up to 86.2 mg/L [110]. Therefore, GEMs are not only essential tools for elucidating the mechanisms underlying CH_4_ metabolism, but also serve as essential platform technologies that promote efficient and sustainable biological utilization of CH_4_. Their applicability is continually expanding across various fields, including metabolic engineering, optimization of industrial fermentation processes, and the development of carbon-neutral strategies, thereby making them a critical technological support for the high-value conversion of CH_4_ resources.

**Table 3.** Genome-scale models of methanotrophs.

Organism	Model	Genes	Reactions	Metabolites	Applications	Ref.
*Methylotuvimicrobium buryatense* 5GB1	*i*Mb5G(B1)	593	841	1007	Investigating the CH₄ oxidation process using flux balance analysis	[134]
*Methylotuvimicrobium buryatense* 5GB1	*i*Mb5GB1	313	442	403	Optimizing conditions for maximal cell growth	[135]
*Methylotuvimicrobium alcaliphilum* 20Z	*i*IA407	407	396	422	Predicting novel metabolic reactions by controlling carbon flux distribution with computational simulations	[34]
*Methylotuvimicrobium alcaliphilum* 20Z		405	437	429	Identifying potential genes to increase metabolic flux towards isoprenoid production	[136]
*Methylotuvimicrobium alcaliphilum* 20Z	*i*20ZR-BDO	325	437	419	Performing *in silico* gene knockout predictions to couple cell growth with 2,3-BDO production	[110]
*Methylococcus capsulatus* Bath	*i*McBath	730	913	879	Simulating CH_4_ oxidation pathways to optimize SCP production	[137]
*Methylococcus capsulatus* Bath	*i*MC535	535	899	865	Investigating the essentiality of the ED pathway for C1 assimilation	[138]
*Methylocystis* sp. SC2		2251	1449	1434	Exploring the redox-arm mechanism in driving CH_4_ oxidation	[139]
*Methylocystis* sp. SB2		2281	1380	1453	Exploring the redox-arm mechanism in driving CH_4_ oxidation	[139]
*Methylocystis parvus* OBBP		2795	1326	1399	Predicting biomass yield on CH_4_	[140]
*Methylocystis hirsuta* CSC1		2748	1350	1428	Exploring the redox-arm mechanism in driving CH_4_ oxidation	[140]
*Methylosinus trichosporium* OB3b	*i*MsOB3b	683	1040	1020	Investigating the redox-arm mechanism	[141]
*Methylosinus trichosporium* OB3b	*i*MsOB3b_cadaverine	686	1046	1025	Predicting metabolic flux distributions for cadaverine and lysine production, and identifying metabolic engineering targets to enhance their yields	[142]

## MICROBIAL PRODUCTION OF ORGANIC MOLECULES FROM METHANE

CH_4_, recognized as a plentiful and cost-effective carbon source, presents significant opportunities for applications in energy and raw materials. With the increasing demand for environmental protection and sustainable development, the efficient utilization of CH_4_ has emerged as a critical area of focus within both scientific inquiry and industrial practice. Recent advancements have highlighted various methodologies involving methanotrophic bacteria, which facilitate the conversion of CH_4_ to key intermediates such as methanol, formaldehyde, and formate. Furthermore, these intermediates can be further transformed into functional chemicals, including organic acids, single-cell proteins, biomaterials, biofuels, and other natural products (Table 4). The development of these bio-based products not only enhances the utilization efficiency of CH_4_ resources but also paves the way for innovations in green chemistry and bioenergy development.

**Table 4.** Microbial production of compounds.

Chemicals	Hosts	Production	Metabolic engineering strategies	Ref.
Organic acids				
Lactic acid	*M. buryatense* 5GB1C	0.8 g/L, 0.05 g/g CH_4_, 0.008 g/L/h	• L-lactate dehydrogenase overexpression • Fermentation engineering	[108]
Lactic acid	*M. buryatense* 5GB1C	0.14 mmol/gDCW/h	• A small-scale cultivation platform • Synthetic biology approach	[107]
Lactic acid	*M. alcaliphilum* 20Z	0.027 g/gDCW/h	• Continuous gas fermentation	[143]
Lactic acid	*Methylomonas* sp. DH-1	1.19 g/L, 0.245 g/g CH_4_	• ALE • Genome sequencing • Metabolic engineering	[33]
Crotonic acid	*M. buryatense* 5GB1C	70 mg/L	• Metabolic engineering • RBS engineering	[37]
Butyric acid	*M. buryatense* 5GB1C	40 mg/L	• Metabolic engineering • RBS engineering	[37]
Muconic acid	*M. buryatense* 5GB1C	12.4 mg/L, 1.2 mg/g CH_4_, 0.023 mg/gDCW/h	• GEM • Weakening the competing pathway • Continuous gas fermentation	[144]
Muconic acid	*M. alcaliphilum* 20ZR	0.75 mg/L, 0.65 mg/g CH_4_, 0.025 mg/gDCW/h	• GEM • Weakening the competing pathway • Continuous gas fermentation	[144]
Muconic acid	*M. capsulatus* bath	0.97 mg/L, 2.8 mg/g CH_4_, 0.014 mg/gDCW/h	• GEM • Weakening the competing pathway • Continuous gas fermentation	[144]
Succinic acid	*Methylomonas* sp. DH-1	195 mg/L, 0.0789 g/g CH_4_	• Blocking the competing pathway • Metabolic engineering	[31]
4-Hydroxybutyric acid	*M. trichosporium* OB3b	10.5 mg/L	• Reconstructing the biosynthetic pathways	[145]
3-Hydroxypropionic acid	*M. trichosporium* OB3b	60.59 mg/L	• Enhancing precursor supply • Refactoring bypass pathways	[18]
Fatty acids	*M. buryatense* 5GB1C	111 mg/L	• RNA-seq analysis	[125]
Fatty acids	*M. buryatense* 5GB1C	108.2 mg/gDCW	• Metabolic engineering	[146]
				
Single-cell proteins				
Single-cell protein	*M. capsulatus* Bath	0.64 g/g CH_4_	• Fermentation engineering	[147]
Single-cell protein	*M. capsulatus* Bath	70%	• Gas fermentation technology	[148]
Single-cell protein	*M. capsulatus* MIR	71%	• Batch and continuous cultivation • Genome analysis	[149]
Biomaterials				
PHB	*M. trichosporium* OB3b	48.7 mg/L	• Statistical regression analysis • RSM	[150]
PHB	*Methylocystis* sp. GB 25 DSMZ 7674	0.55 g/g CH_4_	• Batch cultivation	[151]
PHB	*M. parvus* OBBP	68% w/w	• Fed-batch cultivation • Enrichment strategy	[152]
1,2-Propanediol	*M. trichosporium* OB3b	251.5 mg/L	• Screening the optimal enzymes • Metabolic engineering	[47]
Poly-β-hydroxybutyrate	*M. trichosporium* OB3b	48.7 mg/L, 52.5% DCW	• Statistical regression analysis with interactions • RSM • GEM	[150]
Ectoine	*M. alcaliphilum* 20Z	16.5–37.4 mg/g wet cells	• Fed-batch cultivation	[153]
Ectoine	*M. alcaliphilum* 20Z	9.1 mg/g wet cells	• BLAST • Batch cultivation • Metabolic engineering	[154]
Hydroxyectoine	*M. alcaliphilum* 20ZDP	22.2 mg/g wet cells	• Blocking the accumulation of by-products • Batch cultivation	[16]
ɑ-Humulene	*M. alcaliphilum* 20Z	0.75 mg/g DCW	• Metabolic engineering • GEM • Codon optimization	[136]
Shinorine	*M. alcaliphilum* 20Z	17.13 mg/L, 3.99 mg/g DCW	• Metabolic engineering • Refactoring bypass pathways	[18]
				
Biofuels				
2,3-Butanediol	*M. alcaliphilum* 20Z	86.2 mg/L, 0.0318 g/g CH_4_	• Metabolic engineering • Screening promoters • Optimizing translation initiation rates • GEM	[110]
Lipids	*M. buryatense* 5GB1	45.4 mg/L/h	• Continuous gas fermentation • High cell density culture	[155]
Methanol	*Methylomonas* sp. DH-1	1.34 g/L	• Fed-batch cultivation	[13]
Methanol	*M. trichosporium* OB3b	1.1 g/L	• Membrane aerated reactor	[156]
Methanol	*M. bryophila*	4.63 mM	• Fed-batch cultivation • Optimizing fermentation conditions	[157]
Methanol	*M. sporium*	6.12 mM	• Optimizing fermentation conditions • Batch cultivation	[158]
Natural products				
Putrescine	*M. alcaliphilum* 20Z	98.08 mg/L	• ALE • Blocking downstream pathways • GEM • Transcriptome analysis	[159]
Astaxanthin	*Methylomonas* sp. 16a	2.4 mg/g DCW	• Tuning gene copy number • Genetic engineering	[160]
Astaxanthin	*Methylomonas* sp. 16a	2 mg/g DCW	• Screening chromosomal locations • Metabolic engineering	[161]
Cadaverine	*M. trichosporium* OB3b	283.63 mg/L	• Metabolic engineering • Optimizing metabolic pathway	[120]
Taxadiene	*M. buryatense 5GB1C*	104.88 mg/L	• Metabolic engineering • Screening promoters • Batch cultivation	[162]

### Organic acids

Organic acids represent significant products derived from CH_4_ bioconversion, with wide applications in the chemical, food, and pharmaceutical industries, thereby possessing considerable industrial value. Through the detailed analysis and engineering of the metabolic pathways of methanotrophs, artificial methanotrophic cell factories have been successfully developed that can efficiently convert CH_4_ to various short- and medium-chain organic acids, such as lactic acid, crotonic acid, muconic acid, and succinic acid. Recent advancements in the integration of metabolic engineering, synthetic biology, and systems biology tools have markedly improved the metabolic flux through CH_4_ assimilation pathways, facilitating the value-added utilization of CH_4_ and presenting novel strategies for the biosynthesis of specific target compounds. For example, the heterologous expression of lactate dehydrogenase (LDH) from *Lactobacillus helveticus* in *M. buryatense* facilitated the production of lactic acid from CH_4_ with a titer of 0.06 g/L. Subsequent optimization of the cultivation conditions in a 5-L bioreactor, lactic acid production was further increased to 0.8 g/L, with a productivity of 8 mg/L/h [108]. Furthermore, the introduction of heterologous metabolic pathways into *M. buryatense* allowed for the biosynthesis of other organic acids such as crotonic acid and butyric acid from CH_4_. By screening and optimizing functional genes *atoB* (thiolase), *fadB* (3-hydroxybutyryl-CoA dehydrogenase), and *ydiI* (thioesterase) from various host organisms, the production of crotonic acid and butyric acid from CH_4_ in *M. buryatense* 5GB1C reached 70 mg/L and 40 mg/L, respectively [37]. In *M. buryatense* 5GB1C, deletion of the genes *sps* (sucrose-phosphate synthase), *glgA1* (glycogen synthase), and *glgA2* (glycogen synthase), combined with the overexpression of acetyl-CoA carboxylase, resulted in a fatty acid content up to 108.2 mg/gDCW [146]. Additionally, the heterologous expression of dihydroxyshikimic acid dehydratase (AsbF) from *Bacillus thuringiensis*, protocatechuate decarboxylase (AroY) from *Enterobacter cloacae*, and catechol dioxygenase variant CatAP76A from *Acinetobacter* sp. in *M. buryatense* 5GB1 enabled the conversion of the shikimate pathway intermediate dihydroxyshikimic acid into muconic acid. Through pathway engineering, the maximum yield of muconic acid using CH_4_ as the sole carbon source reached 2.8 mg/g CH_4_ [144]. In summary, methanotrophs, through metabolic engineering strategies, demonstrate significant capability in the efficient synthesis of a diverse array of organic acids, underscoring their great potential as platform organisms for CH_4_ bioconversion in green chemistry and sustainable biomanufacturing.

### Single-cell proteins

Single-cell proteins (SCPs) refer to protein-rich biomass derived from microorganisms such as bacteria, yeast, and algae, and is recognized as a viable alternative protein source for food, animal feed, and industrial applications. Methanotrophs, a specific group of microorganisms capable of using CH_4_ as their sole carbon and energy source, have gained prominence as promising candidates for SCP production due to their rapid growth rates, high protein content, and the cost-effectiveness and abundance of CH_4_. These microbial cell factories present innovative strategies for the efficient utilization of CH_4_ resources, while simultaneously contributing to greenhouse gas mitigation, resource recycling, and sustainable bioconversion processes. To date, various methanotroph strains have been developed for SCP production. For example, *M. capsulatus* Bath has been extensively investigated for its remarkable efficiency in producing SCP from CH_4_, exhibiting advantageous traits such as amino acid composition, digestibility, positive effects on animal productivity, and health outcomes. In mixed bacterial cultures cultivated on CH_4_, crude protein accounted for 71% of the cell dry weight, with a protein yield of 0.64 g/g CH_4_ [147]. Furthermore, *M. capsulatus* Bath has been commercialized as a model microorganism by companies such as Calysta and Unibio for incorporation into animal feed, with a protein content of up to 70% of dry cell weight, and is noted for its richness in essential amino acids and has high nutritional value [148]. In addition, construction of a Δ*glgA1*Δ*glgA2* glycogen synthase mutant of *M. capsulatus* MIR has resulted in a mutant strain with an enhanced protein content, reaching 71% of dry cell weight, thereby significantly augmenting the yield of SCP [149]. In summary, methanotrophs represent a pivotal platform for the biosynthesis of SCP from CH_4_, showcasing not only superior metabolic efficiency and nutritional quality but also providing a practical and feasible technological route for the high-value conversion of CH_4_ and the sustainable production of protein. However, in addition to nutritional value and production efficiency, the safety and toxicological properties of SCP are essential considerations when it comes to its widespread application. Previous research and commercial practices have demonstrated that SCP products derived from CH_4_-oxidizing bacteria, such as *M. capsulatus* Bath, are generally safe for animal feed, showing no significant toxicity or harmful metabolites [163,164]. Nevertheless, potential risks such as endotoxins (e.g. lipopolysaccharides), residual nucleic acids, and possible allergens necessitate thorough toxicological assessments, animal feeding trials, and long-term intake studies to ensure safety [165,166]. These comprehensive safety evaluations not only validate the feasibility of SCPs in food and feed applications but also lay the foundation for its broader implementation on a large scale.

### Biomaterials

Biomaterials represent a category of materials synthesized by living organisms, characterized by their superior biocompatibility, biodegradability, and functional properties. These materials find extensive applications across various fields such as medicine, agriculture, and environmental science. With the growing emphasis on sustainable development, the synthesis of biomaterials from renewable carbon sources has emerged as a prominent area of research. Among these, CH_4_, an economical and carbon-dense feedstock, can be metabolically converted by methanotrophic bacteria into various high-value biomaterials, such as polyhydroxyalkanoates (PHAs), polyhydroxybutyrate, and isoprene. Recent advancements in synthetic biology and metabolic engineering have facilitated the design and optimization of biosynthetic pathways for biomaterial production in various methanotrophs. These efforts have systematically validated the potential for generating functional biomaterials using CH_4_ as the sole carbon source, thereby laying both theoretical and practical groundwork for the development of sustainable biomaterials derived from C1 resources. PHAs are natural polymers synthesized and accumulated by various microorganisms under conditions of surplus carbon sources coupled with nutrient limitations such as nitrogen or phosphorus. In natural ecosystems, these polymers can be biodegraded by microorganisms into CO_2_ and water. Among the different types of PHAs, poly(3-hydroxybutyrate) (PHB) is the most prevalent, exhibiting physical properties similar to polypropylene, and has been extensively investigated for applications in biodegradable packaging materials. In *M. trichosporium* OB3b, the production conditions for PHB were optimized through statistical regression analysis and response surface methodology, resulting in a PHB concentration of ∼48.7 mg/L, which constituted 52.5% of the cell dry weight [150]. Isoprene, a widely studied volatile five-carbon hydrocarbon, is another extensively researched compound that serves as a crucial platform chemical for the synthesis of polyisoprene, elastomers, synthetic rubber, adhesives, and pharmaceuticals. In *Methylocystis* sp. GB 25 DSMZ 7674, the PHB content reached up to 51% of the cell dry weight when cultured with CH_4_ as the sole carbon source, achieving a maximum yield of ∼0.55 g/g CH_4_ [151]. In summary, methanotrophs not only possess the ability to convert greenhouse gases into functional materials but also present a novel platform for developing sustainable material synthesis systems based on C1 resources.

### Biofuels

Biofuels represent a category of high-value metabolic products derived from methanotrophic bacteria. As a critical component of sustainable energy systems, biofuels have attracted widespread attention due to their carbon-neutral potential and diverse feedstock sources. Among these biofuels, biologically synthesized CH_4_ is particularly important for the advancement of biofuel technologies. In 2013, the U.S. Department of Energy’s Advanced Research Projects Agency–Energy (ARPA-E) initiated a program entitled ‘Reducing Emissions using Methanotrophic Organisms for Transportation Energy’ (REMOTE) to expedite the development of economically viable biofuel production technologies [167]. To date, the primary biofuels synthesized from methanotrophic bacteria include 2,3-butanediol, isobutanol, and lipids. Among these biofuels, lipids are essential precursors for the production of green diesel and are predominantly sourced from the intracellular membrane system. Recent advancements in bioconversion technologies have enabled the successful engineering of *M. buryatense* to convert CH_4_ to lipids, which can then be catalytically upgraded to produce diesel-range hydrocarbons suitable for biofuel applications [168]. Investigations into the mechanism of lipid biosynthesis, *M. buryatense* 5GB1 and its engineered variant AP18 (5GB1CΔ*glgA1*Δ*glgA2*Δ*sps*) have provided extensive characterization of these strains. The findings indicate that their membrane lipids are primarily composed of fatty acids such as myristic acid, palmitic acid, and palmitoleic acid. By optimizing fermentation conditions through high-cell-density cultivation and continuous aeration strategies, the maximum cell dry weight of *M. buryatense* 5GB1 reached 21.4 g/L, with a peak lipid synthesis rate of 45.4 mg/L/h. These results highlight the strain’s considerable potential for the biosynthesis of precursors for green diesel production [155]. In summary, biological CH_4_ synthesis is not only integral to biofuel systems but also serves as a critical pathway for fostering a circular economy and achieving concurrent energy and environmental optimization. Looking ahead, the integration of synthetic biology and systems bioengineering is poised to significantly enhance microbial energy conversion efficiency, thereby promoting the industrialization and diversification of biofuels.

### Natural products

With the advancement of metabolic engineering and synthetic biology, CH_4_ bioconversion is becoming an important technological approach for producing high-value natural products across various fields such as medicine, nutrition, and agriculture. The scope of its applications has broadened beyond conventional products such as organic acids, SCPs, biomaterials, and biofuels to encompass a wider array of products. Notably, the overexpression of aspartokinase (LysC) and diaminopimelate decarboxylase (LysA) in *M. trichosporium* OB3b, in conjunction with the heterologous introduction of pyruvate carboxylase (Pyc) from *Methylomonas* sp. DH-1 and lysine–cadaverine antiporter (CadB) from *E. coli*, resulted in cadaverine production of 283.63 mg/L in a gas-phase bioreactor [121]. Furthermore, the heterologous expression of ornithine decarboxylase from *M. trichosporium* OB3b in *M. alcaliphilum* 20Z facilitated the production of up to 12.44 mg/L of putrescine. Subsequent enhancements through genome-scale metabolic modeling and optimized cultivation conditions elevated putrescine production to 98.08 mg/L [159]. In addition, various natural products such as limonene, farnesene, torularhodin, lycopene, and astaxanthin have been successfully synthesized from CH_4_ in methanotrophs. For example, in *Methylomonas* sp. strain 16a, which uses CH_4_ as the sole carbon source, genetic engineering was applied to control the copy numbers of the *crtW* (β-carotene ketolase) and *crtZ* (β-carotene hydroxylase) genes, which encode β-carotenoid ketolase and hydroxylase, respectively, resulting in astaxanthin production up to 2.4 mg/g dry cell weight [160]. In summary, CH_4_ bioconversion is progressively extending its application frontiers in the synthesis of functional natural products, thereby offering novel carbon feedstocks and technological pathways for high-value biomanufacturing.

## CONCLUSION AND OUTLOOK

CH_4_, a major component of greenhouse gases, is a low-cost, energy-rich carbon source [16]. It can serve as a sustainable raw material for biofuels and biochemicals, offering significant potential for the development of a low-carbon economy [136]. Three types of methanotrophs are capable of utilizing CH_4_ as a carbon and energy source, positioning themselves as pivotal players in the study of CH_4_ bioconversion. These microorganisms achieve CH_4_ utilization through the RuMP cycle, serine cycle, and CBB cycle [169]. In addition, the efficiency of CH_4_ bioconversion can be improved by many metabolic engineering and synthetic biology strategies, such as developing genetic modification tools, enhancing CH_4_ utilization pathways, regulating CH_4_-utilizing cells adaptability, and omics-driven strategies. As an environmentally friendly approach to mitigate climate change, CH_4_ bioconversion holds considerable promise [170]. Nevertheless, the bioconversion of CH_4_ remains at an early stage and is constrained by biological, technological, and economic factors. Its industrial implementation is still limited, impeding the efficient utilization of methane and posing substantial challenges to the advancement of a CH_4_-based economy. Future development can be structured around three key objectives, progressively promoting a highly efficient and sustainable CH_4_ bioeconomy.

The goal of the first stage is to gain an in-depth understanding of the physiological characteristics of methanotrophs and the mechanisms of CH_4_ metabolism. Currently, there are primarily two limiting factors. The first pertains to the inherent limitations of microorganisms themselves. There exists a paucity of biological information regarding methanotrophs. Despite various omics technologies having been used to characterize them, their protein functions and metabolic pathways have not yet been elucidated [125,127]. To strengthen the systematic understanding of methanotrophs, a genome-scale metabolic network model can be constructed to analyze the metabolic mechanism of methanotrophs from a broader perspective and provide a foundation for exploring CH_4_ utilization. In addition, artificial intelligence (AI)-assisted protein structure prediction and directed evolution can be employed to optimize MMO function, while improving CH_4_ mass transfer efficiency to alleviate solubility limitations. At the same time, the key enzyme MMO for CH_4_ bioconversion cannot be expressed in other host strains, which hinders its industrial application [171]. Thus, addressing this challenge by analyzing MMO structure is crucial, as the exploration of alternative enzymes for MMO can facilitate the heterologous expression of the CH_4_ assimilation pathways. The second limiting factor lies in the limitations of genetic manipulation tools. Due to the above-mentioned challenges associated with MMO, the CH_4_ conversion pathways cannot be effectively applied in traditional model microorganisms (such as *E. coli* and *S. cerevisiae*), thereby precluding the use of established manipulation tools typically available for these model microorganisms in CH_4_ research. Furthermore, the development of gene editing tools for methanotrophs remains inadequate and insufficient for industrial applications [107,111,112]. Thus, developing genetic modification tools tailored for methanotrophs is of paramount importance.

The focus of the second stage is to optimize CH_4_ conversion efficiency through metabolic engineering and synthetic biology. For CH_4_ utilization pathways, the limiting factors mainly stem from the complexity of these pathways, the specificity of pathway enzymes, and their inefficiency in CH_4_ assimilation. Currently, there are only three natural CH_4_ assimilation pathways, all of which are involved in complex reactions. For example, the RuMP pathway consists of three stages: carbon fixation, cleavage, and rearrangement, and its metabolic efficiency remains suboptimal [100]. Optimizing these pathways necessitates a comprehensive consideration of carbon flux from a holistic perspective rather than merely focusing on a single two-step reaction. Second, the enzymes in the CH_4_ utilization pathway are specific and often exhibit poor stability and low efficiency. For example, MMO is a unique enzyme that cannot be expressed in heterologous systems, but the addition of formate has been shown to increase MMO activity [117]. By precisely controlling the CH_4_/O_2_ ratio, the sensor feedback system can maintain an optimal oxygen concentration, thereby balancing metabolic requirements with MMO activity. Moreover, there has yet to emerge an artificially designed CH_4_ assimilation pathway. Notably, the integration of AI with systems biology is opening new avenues for the optimization of CH_4_ assimilation pathways. Deep learning-based protein structure prediction platforms, such as AlphaFold2, facilitate precise modeling of key enzymes, thereby providing a solid foundation for advancing heterologous expression, directed evolution, and catalytic improvement [172]. In addition to enzyme-level optimization, AI-guided exploration of metabolic pathways and design of synthetic networks offer the potential to reconstruct existing or even create novel CH_4_ assimilation routes. This approach helps overcome the intrinsic inefficiencies of natural pathways and broadens the scope of metabolic engineering possibilities [173]. Thus, both the reconstruction and the artificial design of streamlined pathways for CH_4_ utilization represent effective strategies for optimizing CH_4_ bioconversion. Through metabolic engineering and synthetic biology, CH_4_ assimilation pathways can be reconstructed or artificially designed to optimize carbon flux distribution and regulate strain adaptability, thereby addressing oxygen dependency and product inhibition issues and achieving efficient CH_4_ utilization.

For microbial engineering strategies, the main considerations are product inhibition and oxygen dependence. During CH_4_ bioconversion, certain metabolites may inhibit the growth of microorganisms. For instance, the high concentration of lactic acid can affect the growth of *Methylomonas* sp. DH-1JHM80. Gene expression regulation strategies such as introducing exogenous lactate dehydrogenase and knocking out glycogen synthase can optimize CH_4_ utilization and increase lactic acid production [33]. On the other hand, CH_4_ oxidation typically requires oxygen as an electron acceptor, but excessive oxygen concentrations can inhibit the activity of MMO, thereby affecting CH_4_ utilization [174,175]. How to accurately control the ratio of oxygen and CH_4_ is a challenge, as it is essential to provide sufficient oxygen to support metabolic processes without excessively inhibiting the activity of key enzymes. Thus, to improve the ability to utilize CH_4_, these limiting factors must be fully considered and balanced to achieve optimal performance.

The final stage aims to advance CH_4_ bioconversion from laboratory research to industrial-scale application. Technical economic analysis (TEA) and life cycle assessment (LCA) play an important role in assessing the economic feasibility and environmental impact of CH_4_ bioconversion. TEA indicates that the cost-effectiveness of CH_4_ bioconversion is mainly affected by many factors such as CH_4_ source (such as natural gas and biogas), gas-liquid mass transfer efficiency, target product yield, and downstream extraction and purification costs. Different technical routes exhibit different potential in the commercialization of CH_4_ bioconversion. Natural methanotrophic hosts, represented by CH_4_-oxidizing bacteria such as *M. capsulatus*, have exhibited high feasibility for industrial production of SCPs [148]. Currently, representative target products include SCPs, organic acids (such as lactic acid and acetic acid), and biofuels [147]. Despite its promising potential, CH_4_ bioconversion still faces many challenges: the inefficiency of gas-liquid mass transfer limits CH_4_ utilization; the robustness of the strain under high CH_4_ concentrations and complex environmental conditions still needs to be improved; high costs associated with the downstream separation and purification processes require urgent resolution. To address these challenges, future efforts should focus on increasing strain production capacity through metabolic engineering and optimizing reactor design to improve gas transfer efficiency, thereby reducing overall production costs [121]. LCA shows that when CH_4_ is used as raw material, CH_4_ bioconversion can significantly reduce greenhouse gas emissions and fossil energy consumption, compared to traditional pathways, thereby offering substantial environmental benefits. Efficient CH_4_ utilization not only helps to alleviate its greenhouse effect potential, but also promotes transformation of the carbon cycle into a low-carbon and sustainable trajectory [176]. Overall, CH_4_ bioconversion has great potential for achieving economic feasibility and environmental friendliness by leveraging low-carbon CH_4_ sources and optimizing process design.

For popularization and application, there is a gradual establishment and enhancement of the relevant legal and regulatory frameworks. Globally, many countries have enacted policies to promote the utilization of low-carbon resources such as biogas and recoverable urban CH_4_. For example, the European Union’s Renewable Energy Directive (RED II) underscores the significance of CH_4_ recovery in mitigating greenhouse gas emissions and facilitates its advancement through tax incentives and carbon credit systems [177]. In addition, the industrialization of CH_4_ bioconversion must adhere to existing regulations concerning environmental protection, safety, and biotechnology. Consequently, it is imperative to actively advocate for regulatory updates and to strengthen policy support mechanisms in order to achieve the sustainable implementation of these initiatives in the transition towards a green and low-carbon economy.
